# Coupling Electrochemical CO_2_ Reduction With Ethanol Oxidation for Acetate Production in a Dual‐Electrolyzer System

**DOI:** 10.1002/anie.9339732

**Published:** 2026-04-02

**Authors:** Anirudha Shekhawat, Shubhadeep Chandra, Ridha Zerdoumi, Bashir Eid, Muhammad Adib Abdillah Mahbub, Wolfgang Schuhmann

**Affiliations:** ^1^ Analytical Chemistry‐Center for Electrochemical Sciences (CES) Faculty of Chemistry and Biochemistry Ruhr University Bochum Bochum Germany

**Keywords:** acetate, CO_2_RR, coupled electrolysis, ethanol oxidation, tandem cell

## Abstract

Bioethanol production from biomass is suggested as a strategy for greener fuel production. However, an equimolar amount of CO_2_ is released during fermentation, making the process less efficient. Utilizing this released CO_2_ directly in an electrolyzer can further reduce carbon emissions, ultimately reaching a CO_2_‐negative carbon balance. We coupled the electrochemical CO_2_ reduction reaction with ethanol oxidation as the anode reaction for acetate production. A nickel foam‐supported cobalt selenium catalyst, which is highly active for ethanol oxidation, reached a Faradaic efficiency of nearly 96% towards acetate. A high production rate of 95.2 µmol cm^−2^ min^−1^ at a current density of 1 A cm^−2^ in a model flow‐through electrolyzer was achieved. For coupled electrolysis at 100 mA cm^−2^, we achieved 99% and 95% FE for CO and acetate at the cathode and anode, respectively. Furthermore, using a defect‐Cu‐triazole catalyst on the gas diffusion cathode, we show a tandem cell system coupled with ethanol oxidation in both reactors for selective acetate production. This proof‐of‐concept approach can be further developed for a greener approach to bioethanol and acetate production.

## Graphical abstract

A tandem cell system coupling electrochemical CO_2_ reduction and electrochemical ethanol oxidation is demonstrated for overall acetate production, providing concepts for a CO_2_‐negative bioethanol to acetate pathway.

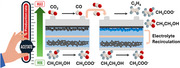

## 1 Introduction

Bioethanol has emerged as a viable alternative to fossil fuels as the world's energy demands continue to rise [1]. Bioethanol is a sustainable fuel made from biomass that can help reduce greenhouse gas emissions [2]. It also plays a vital role in decarbonizing the transportation sector, as ethanol‐based fuels are increasingly used to meet sustainability targets and environmental requirements [3, 4].

Bioethanol is primarily produced through microbial fermentation of biomass feedstocks such as corn, sugarcane, and lignocellulose from agricultural products or residues [5, 6]. Several energy‐intensive steps are involved in the production process, such as biomass pretreatment to increase enzyme accessibility, enzymatic hydrolysis that releases fermentable sugar, microbial fermentation to convert these sugars into ethanol, and downstream purification steps like distillation and dehydration [7, 8]. However, significant carbon losses occur during fermentation with microorganisms like Saccharomyces cerevisiae, which are breaking down carbohydrates into ethanol and carbon dioxide (CO_2_) in a stoichiometric 1:1 molar ratio [9]. Hence, bioethanol production emits substantial amounts of CO_2_ and likely loses its carbon neutrality unless it is fuelled by renewable energy sources or combined with carbon capture and storage technologies [10]. The CO_2_ produced during fermentation is highly concentrated and nearly pure, with minor impurities of ethanol, water vapour, and other volatile organic compounds [11]. This high‐purity CO_2_ stream is well‐suited for direct use in the CO_2_ reduction reaction (eCO_2_RR), offering a potential synergy between bioethanol production and electrochemical CO_2_ utilization, therefore eliminating the need for CO_2_ capture, transportation, and emissions‐related concerns for this process.

The obtained bioethanol can be further utilized to produce acetate [12]. Coupling eCO_2_RR with different anodic reactions is gaining more attention. Two different Ni/Fe dual metal‐atom catalysts were synthesized and coupled with eCO_2_RR and hydroxymethylfurfural oxidation to produce CO and 2,5‐furandicarboxylic acid, respectively [13, 14]. However, targeting a single C_2+_ product from both reactors is challenging, especially acetate, due to high CO* coverage and locally high pH requirements [15]. Furthermore, the electrochemical production of acetate through eCO_2_RR or the oxidation of ethanol (eEOR) can be a promising approach [16, 17].

Recently, Jiao et al. have demonstrated the production of acetate by electrocatalytic CO_2_/CO reduction using a kW‐scale CO electrolyzer stack at a stable current of 300 A over 125 h, yielding 98 L of 1.2 M acetate at 96% purity [18].

**SCHEME 1:**
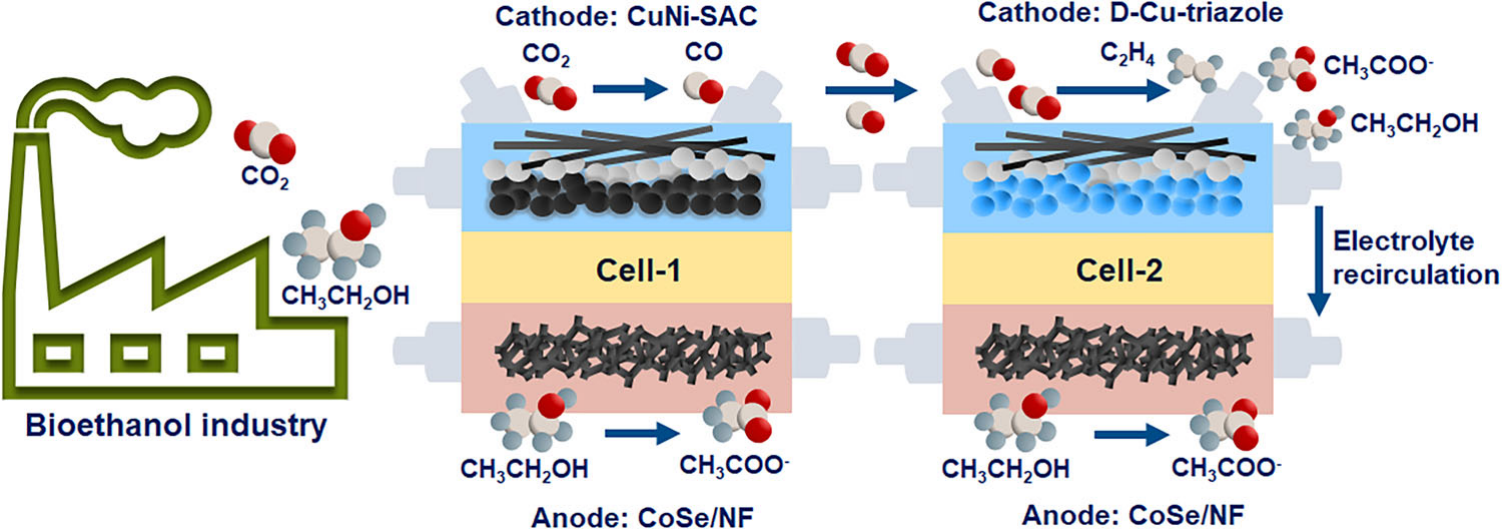
Conceptual scheme for a coupled tandem cell system for ethanol oxidation and CO_2_ reduction. The acetate yield is enhanced by recirculating the ethanol produced during eCO_2_RR into the anodic compartment for further oxidation to acetate.

We demonstrate a proof‐of‐concept system, using a tandem flow‐through electrolyzer system integrating eCO_2_RR with eEOR to enhance overall acetate production at high current densities, using CO_2_ and ethanol (Scheme 1). We report a Ni foam modified with a cobalt selenium pre‐catalyst (CoSe/NF) for eEOR, achieving 95.2 µmol cm^−2^ min^−1^ at a current density of 1 A cm^−2^. The tandem electrolyzer uses defect‐rich Cu‐triazole catalysts for eCO_2_RR with electrolyte recirculation to the anode to increase the acetate production rate.

## 2 Results and Discussion

### 2.1 Half‐Cell Measurements eEOR

**FIGURE 1:**
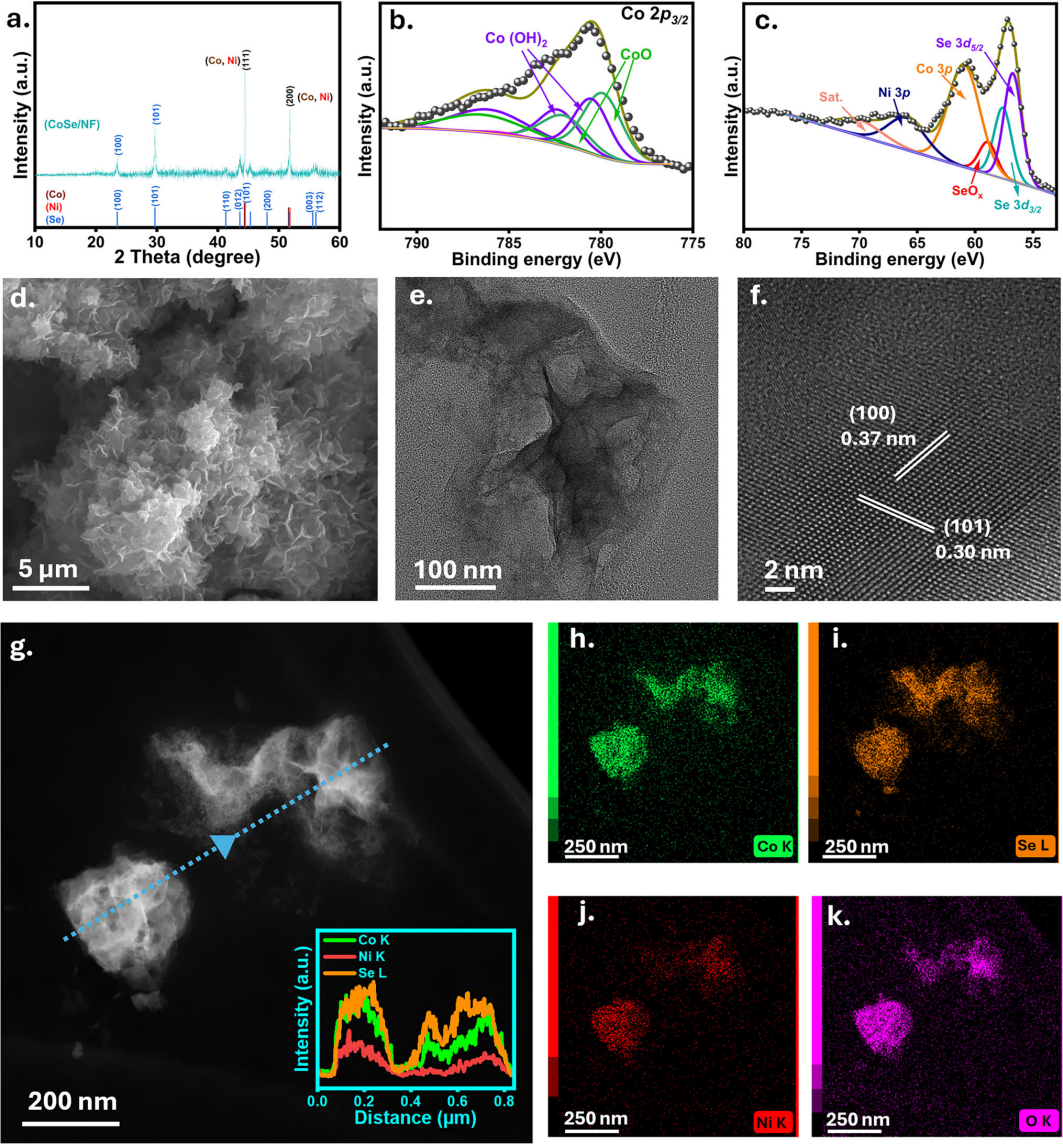
a) p‐XRD patterns of Ni foam modified with the CoSe catalyst together with the theoretical patterns of Co, Ni, and Se. Deconvoluted high‐resolution XPS spectra of b) Co 2*p* and c) Se 3*d*. d) SEM image, e) TEM image, f) HR‐TEM image of the CoSe/NF catalyst. g) STEM image with EDX line scan Co (green), Se (orange), Ni (red). h–k) Corresponding EDX elemental mapping of the CoSe/NF catalyst.

eEOR can be an alternative for the oxygen evolution reaction (OER) in eCO_2_RR electrolyzers due to its lower overpotential and its selectivity towards value‐added products [19]. However, achieving selective acetate/ acetic acid formation at high current density (>100 mA cm^−2^) is hindered by side reactions, favoring the OER over eEOR [20]. This requires specific electrocatalysts which exhibit high selectivity for acetate formation at high current densities. Metal selenides are highly conductive and possess a tunable crystal and electronic structure [21]. We designed a Ni foam‐supported CoSe‐based catalyst (CoSe/NF) using a modified hydrothermal synthesis strategy (for details, see the SI). The X‐ray diffraction (XRD) pattern of CoSe/NF was compared with the theoretical patterns of Co [22], Se [23], and Ni [24] (Figure 1a), indicating the presence of the hcp phase (*hP*3, space group: 152) from Se and the fcc phase (*cF*4, space group: 225) from Ni and Co. X‐ray photoelectron spectroscopy (XPS) was performed to elucidate the near‐surface chemical composition and oxidation state of the catalyst. The high‐resolution XPS spectrum of the Co 2*p_3/2_* region (Figure 1b) indicates a Co^2+^‐rich oxidation state [25], with atomic ratios of 32.37% and 33.74% for CoO and Co(OH)_2_, respectively (Figure S1). The Se 3*d* spectrum (Figure 1c) shows the presence of a SeO_x_ at around 58.89 eV [26]. The relative atomic concentration of Se and SeO_x_ is 28.7% and 5.15%, respectively (Figure S1). The CoSe/NF morphology was derived using field emission scanning electron microscopy (FE‐SEM) and transmission electron microscopy (TEM). SEM images show a nanoflower and nanoflake‐like morphology (Figure 1d). Low‐resolution TEM images further confirm the nanoflake‐type morphology (Figure 1e). Such a morphology can be advantageous due to an increase of the active surface area, and the exposed edges can increase the number of active sites [27]. High‐resolution TEM images show lattice fringes with a distance of 0.30 nm and 0.37 nm assigned to the (101) and (100) planes, respectively, of the Se nanoparticle (Figure 1f). Energy dispersive X‐ray spectroscopy (EDX) in SEM (Figure S2) and TEM (Figure 1g‐k) analyses confirm the uniform distribution of Co and Se on the Ni foam.

**FIGURE 2:**
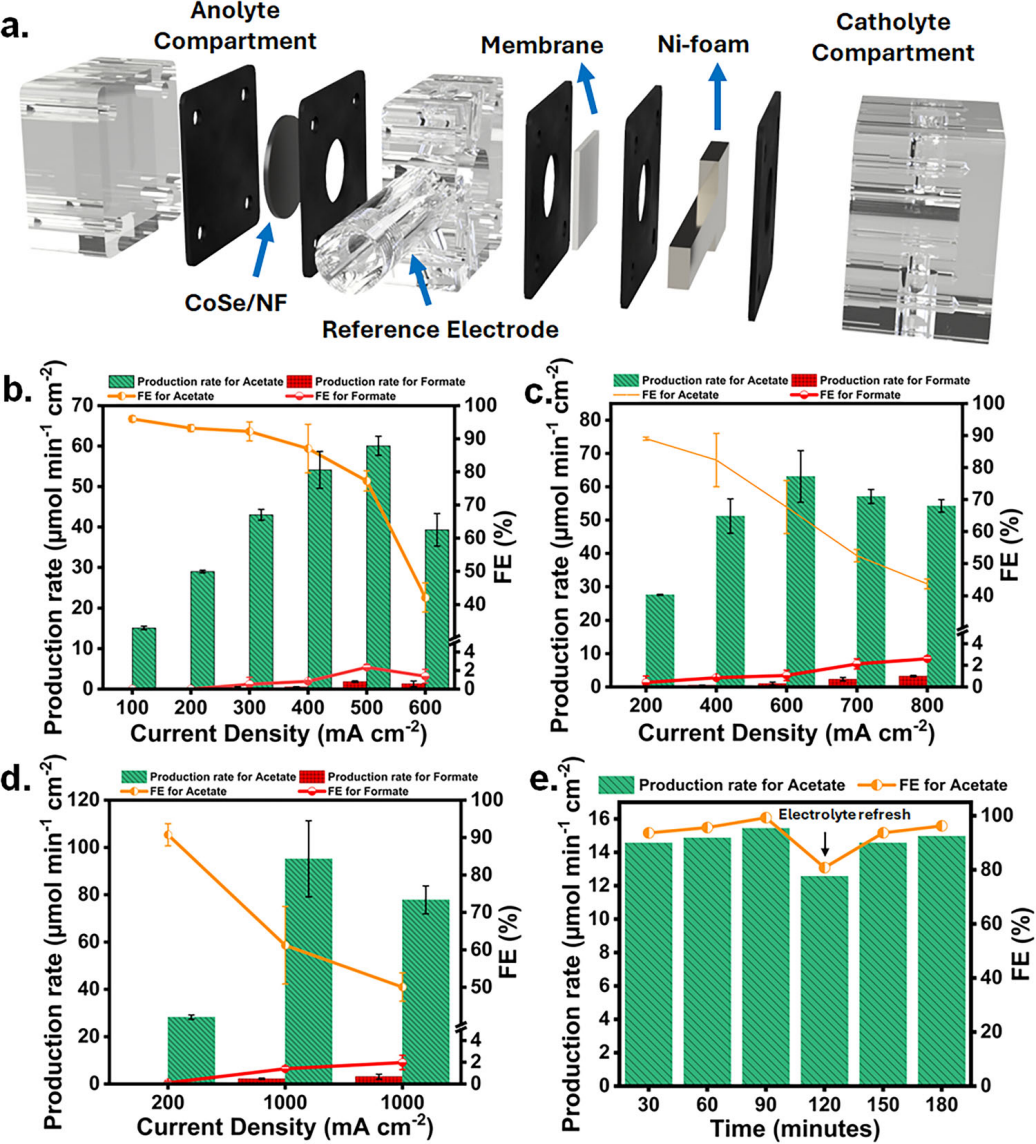
a) Scheme of the flow‐through electrolyzer used for half‐cell eEOR. FE_acetate_ and acetate production rate for the same CoSe/NF electrode in 1 M KOH + 1 M EtOH at different b) current densities, c) after refreshing the electrolyte, d) after refreshing the electrolyte at high current densities. e) FE_acetate_ and acetate production rate during a 3 h long chronopotentiometry measurement at 100 mA cm^−2^ for eEOR.

The eEOR performance of the as‐prepared catalysts was investigated using a three‐electrode model flow‐through electrolyzer (Figure 2a). The scheme of the anodic half‐cell is shown in Figure S3. Pre‐conditioning steps were performed to activate the CoSe/NF catalyst (Figure S4a,b). After that, 5 consecutive linear sweep voltammograms (LSVs) in the potential range from 1.1 V to 1.9 V vs. RHE for OER and EOR. Electrocatalytic activities were evaluated in alkaline electrolyte (1 M KOH) in the presence of varying ethanol concentrations (Figure S4c,d). In the presence of 1 M KOH + 1 M EtOH, CoSe/NF achieved a potential of 1.47 V vs. RHE and 1.58 V vs. RHE at current densities of 50 mA cm^−2^ and 100 mA cm^−2^, respectively. The results show no improvement compared with 1.0 M ethanol concentration.

The oxidation of ethanol involves the co‐adsorption of OH‐ species which may be hindered by surface blocking with ethanol [28]. These potentials are below the overpotentials necessary for the OER. As a control, a bare NF was subjected to the same measurement procedure (Figure S4e,f). A potential of 1.54 V vs. RHE was required at a current density of 50 mA cm^−2^, confirming the improved activity of CoSe/NF for eEOR. We performed galvanostatic measurements at constant current densities of 100, 200, 300, 400, 500, and 600 mA cm^−2^ for 422 s, followed by galvanostatic electrochemical impedance spectroscopy (GEIS) at each current density to determine the uncompensated electrolyte resistance. Figure S5(a,b) shows the average required uncompensated potentials and applied currents during the chronopotentiometric measurements. At 100 mA cm^−2^, the FE for acetate (FE_acetate_) reached 96% with a production rate of 15.12 µmol cm^−2^ min^−1^. Increasing the current density to 500 mA cm^−2^, improved the production rate for acetate to 60.1 µmol cm^−2^ min^−1^, with a FE_acetate_ of nearly 77% (Figure 2b). However, at an even higher current density of 600 mA cm^−2^, the FE_acetate_ decreased to 42.1% with a production rate of 39.3 µmol cm^−2^ min^−1^. This might be due to a substantial local acidification at the electrode/electrolyte interface due to the consumption of OH^−^ in the proton‐coupled electron‐transfer reactions (PCET) during both eEOR and OER.

The depletion of OH^−^ at high current densities creates a gradient that limits the mass transport and charge transfer, and leads to slow reaction kinetics [29]. This effect obviously increases tremendously at high applied current densities and is hence of specific importance in our experiments and is furthermore particularly relevant for electrochemical experiments that utilize recirculation of electrolyte. To confirm this, fresh electrolyte was recirculated in the flow‐through electrolyzer using the same electrode. Chronopotentiometric measurements started at a current density of 200 mA cm^−2^ and continued to 400, 600, 700, and 800 mA cm^−2^. Figure S5c,d shows the average required uncompensated potential for a given applied current density. Interestingly, the catalytic activity was re‐established at 200 mA cm^−2^. The production rate of acetate reached 27.7 µmol cm^−2^ min^−1^ with an FE_acetate_ of 89%, which is comparable to the initial measurement. At 600 mA cm^−2^, the production rate increased from 39.3 to 67.65 µmol cm^−2^ min^−1^, and the FE_acetate_ from 42.1 % to 67.6 % (Figure 2c). After recirculating fresh electrolyte again with the same electrode, measurements were started at current densities of 200, 1000, and again 1000 mA cm^−2^ (Figure 2d). The catalytic activity was again re‐established at 200 mA cm^−2^ with a production rate of 28.2 µmol cm^−2^ min^−1^ and an FE_acetate_ of 90 %.

The production rate increased further to 95.2 µmol cm^−2^ min^−1^ at a current density of 1 A cm^−2^, confirming excellent catalytic activity of the CoSe/NF electrode towards eEOR at high current densities. Figure S5e,f shows the required uncompensated potential for each applied current.

**FIGURE 3:**
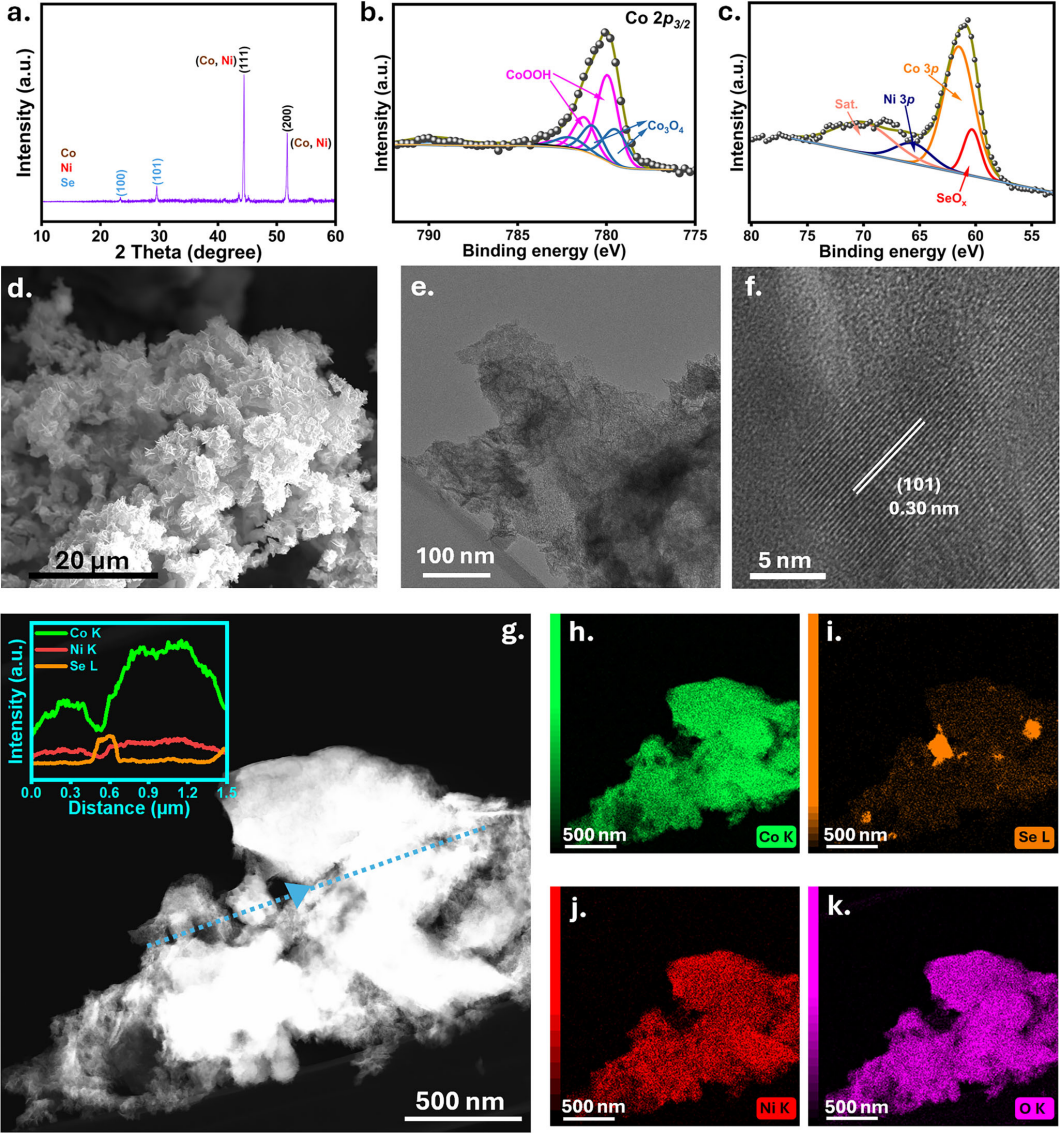
Characterization after of the CoSe/NF catalyst after 3 h long chronopotentiometry measurement at 100 mA cm^−2^. a) p‐XRD patterns of NF modified with the CoSe catalyst. High‐resolution XPS spectra of b) Co 2*p_3/2_* and c) Se 3*d* regions. d) SEM image, e) TEM image, f) HR‐TEM image of the CoSe/NF catalyst after electrolysis. g) STEM image with the EDX line scan of Co (green), Se (orange), Ni (red). h‐k) Corresponding EDX elemental maps of CoSe/NF.

To further investigate the behavior of the CoSe/NF electrode for eEOR, a 3 h measurement at 100 mA cm^−2^ was performed (Figure 2e), showing a similar FE_acetate_ and production rate. The required uncompensated potential and applied current are shown in Figure S6. Post‐electrolysis SEM‐EDX mapping indicates that Se leached out from the catalyst (Figure S7), suggesting that the pristine CoSe/NF acts as a pre‐catalyst that transforms during eEOR. However, despite Se leaching, the catalytic activity remained unaffected, indicating that these structural transformations likely expose more active sites for the eEOR. Previous reports have shown that chalcogens facilitate access to a higher oxidation state of Ni during electrooxidation, enhancing catalytic performance by stabilizing and promoting the formation of reaction intermediates [30]. Additionally, it was suggested that the leached chalcogenate oxyanions can also be re‐adsorbed on the surface of active catalysts during oxidation reactions and act as secondary binding sites, stabilizing reaction intermediates and thereby increasing catalytic activity [31]. Hence, understanding Se leaching during eEOR is crucial for understanding its role in increasing catalytic activity. Post XRD measurements (Figure 3a) show a decrease in the relative intensity of the reflections associated with the *hcp* phase of Se. Furthermore, SEM (Figure 3d) and TEM (Figure 3e) images of the spent catalyst confirmed that the nanoflower and nanoflake‐like morphology was retained. HR‐TEM images (Figure 3f) revealed a d‐spacing value of 0.30 nm corresponding to the (101) plane of Se. However, TEM‐EDX elemental mapping confirmed leaching of Se, suggesting that Co species are exposed upon Se leaching as also further confirmed by the corresponding EDX line scan (Figure 3g–k). These results were further confirmed by XPS measurements after electrolysis (Figure 3b,c). Se was leached out to only 2.8% SeO_x_ (Figure S1). In the Co 3p region, CoOOH dominates (53.1%) over Co_3_O_4_ (38.7%) (Figure S1), demonstrating the continuous transformation of Co to higher oxidation states, and together with ex‐situ XPS, SEM, and TEM measurements indicate that the Se leaching creates a more exposed, Co‐rich surface. Most likely, the corresponding sites have a lower coordination number, facilitating the adsorption of ethanol and oxidized intermediates, further promoting the catalytic reaction [32]. To investigate the structural evolution and surface chemistry of the as‐prepared catalysts during eEOR, we performed operando Raman measurements with gradually increasing applied potentials in alkaline medium (1 M KOH +1 M EtOH) using a custom‐made cell (Figure S8). The prominent Raman bands at 342 cm^−1^ and 817 cm^−1^ can be attributed to the Co‐Se and (SeO_3_) ^2−^stretching vibration, respectively [33]. The characteristic band of ethanol can be observed at 880 cm^−1^ (Figure S9a) [34]. Upon increasing the applied potential, an increase in the vibration band at 417–430 cm^−1^ was observed, suggesting newly formed CoO and Co‐OH representing the CoOOH phase in agreement with the post‐electrolysis XPS measurements (Figure 3b). We observed an adsorbed acetaldehyde intermediate at 706 cm^−1^ (Figure S9a). Quantitative analysis of the Raman bands of the reactant and intermediates during eEOR was performed.

A series of preprocessing steps was necessary to provide comparable Raman spectra. We applied the same baseline subtraction, normalization, and smoothing to all raw spectra (Figure S9b). The band at around 103 cm^−1^ was used as a standard during normalization, corresponding to the characteristic optical fiber band [35]. The intensity of the Raman band at 880 cm^−1^ (I_880_; assigned to ν_*s*_C‐C‐O from ethanol) [36] decreased with increasing potential, suggesting that ethanol is consumed during the reaction. I_420_ and I_706_ (assigned to ν M‐O from Co/Se/Ni‐OOH, M = Co/Se/Ni) slightly increased with I_706_ (adsorbed δ_O‐C‐O_ from acetate) [37, 38], indicating that acetate species are bound to the CoSe/NF surface via the oxygen end of the molecule. These findings are indicative for eEOR with high selectivity towards acetate at CoSe/NF.

### 2.2 Half‐cell Measurements eCO_2_RR

We next focused on the cathodic generation of C_2+_ products, mainly acetate, during eCO_2_RR. We built on a recently described defect‐rich copper triazole‐based (D‐Cu‐triazole) catalyst that exhibited high catalytic activity towards C_2+_ products during eCO_2_RR in alkaline medium in a gas diffusion electrode‐based flow‐through electrolyzer (Figure S10) [39], achieving 79 % FE_C2+_ and a FE of 94% for all eCO_2_RR products at a current density of ‐200 mA cm^−2^ (Figure S11). At a current density of ‐150 mA cm^−2^, this catalyst achieved a FE of 41% towards ethylene and 32% for oxygenated C_2+_ products. Increasing the current density to ‐200 mA cm^−2^ further enhanced the selectivity, achieving a FE of 42% towards ethylene and 37% for oxygenated C_2+_ products, respectively, indicating the catalyst's potential not only for C‐C coupling but also for the direct formation of oxygenated products during eCO_2_RR. Previously, Yang et al. showed that acetate formation is favored at high CO coverage and locally high pH compared to ethylene and ethanol formation [40]. Also, recent work by Wei et al. on coverage‐driven selectivity switch from ethylene to acetate in high‐rate CO_2_/CO electrolysis suggests that by increasing the CO partial pressure in the feed, the major product can gradually switch from ethylene to acetate [41]. By tuning the local reaction environment and increasing CO coverage, the production rate of oxygenated products can be significantly increased. However, optimizing the reaction in a single‐cell eCO_2_RR system is challenging because the C‐C coupling and CO generation take place on the same catalyst surface [42]. To mitigate these limitations, we used a tandem cell setup, where CO_2_ is first reduced to CO (cell‐1) and the resulting gas stream containing CO/CO_2_ is then fed into a second flow‐through electrolyzer for C‐C coupling (cell‐2) [43]. Cell‐1 requires a highly active CO‐producing catalyst to increase the CO/CO_2_ ratio for cell‐2. We previously suggested a 3D microporous carbon CuNi single‐atom catalyst (CuNi‐SAC), which operated at high current densities with exclusive selectivity towards CO during eCO_2_RR [44]. The high catalytic activity for this catalyst is attributed to incorporating Cu in the precursor which modulates the distribution of the N species, resulting in an optimized electronic structure of the Ni‐N_x_ sites.

To ensure a sufficiently CO‐rich stream for cell‐2, we investigated the effect of the CO_2_‐flow rate on the performance of cell‐1. Half‐cell measurements were performed using the setup schematically represented in Figure S12. At a CO_2_‐flow rate of 20 mL min^−1^, current densities of ‐600 mA cm^−2^ with 99% FE_CO_ were reached (Figure S13a). The required uncompensated potential is shown in Figure S13b. The iR‐corrected potential, the applied current, and the GEIS measurements are shown in Figure S13c, respectively. At a CO_2_‐flow rate of 15 mL min^−1^, we saw an increase in H_2_ formation at a current density of ‐500 mA cm^−2^ (Figure S14). Increased flow rates help to sustain a high local CO_2_ concentration by improving the delivery of CO_2_ to the catalyst surface [45, 46]. At a CO_2_‐flow rate of 15 mL min^−1^ and at a current density of ‐400 mA cm^−2^, the demand for CO_2_ increases significantly, and insufficient supply reduce the eCO_2_RR performance. Hence, a CO_2_‐flow rate of 20 mL min^−1^ was chosen for tandem cell measurements. We performed a 1 h measurement of 8 consecutive cycles at a current density of ‐500 mA cm^−2^ (Figure S15), confirming the selectivity of nearly 99% FE_CO_ and stability for at least 8 consecutive cycles.

### 2.3 Paired Electrolysis

The performance of the paired electrolysis was evaluated for cell‐1 using a two‐compartment model electrolyzer as shown in Figure S16a. Recent work discusses about the formation of C_1_ products from coupling eCO_2_RR with different anodic reactions [47, 48]. However, our target was to increase the production rate and selectivity for C_2+_ products. For this, eCO_2_RR‐based electrode was used as the working electrode, while the CoSe/NF electrode served as the counter electrode. Since the formation of eCO_2_RR products, especially C_2+_ products, is potential‐dependent, we used eCO_2_RR as the working electrode. Hence, CuNi SAC was used as a cathode catalyst, and CoSe/NF was used as an anode catalyst. We performed 16 consecutive cycles at ‐100 mA cm^−2^ for 2 h (Figure S16b), yielding nearly 99% FE_CO_ and 95% FE_acetate_, respectively (Figure S16c).

**FIGURE 4:**
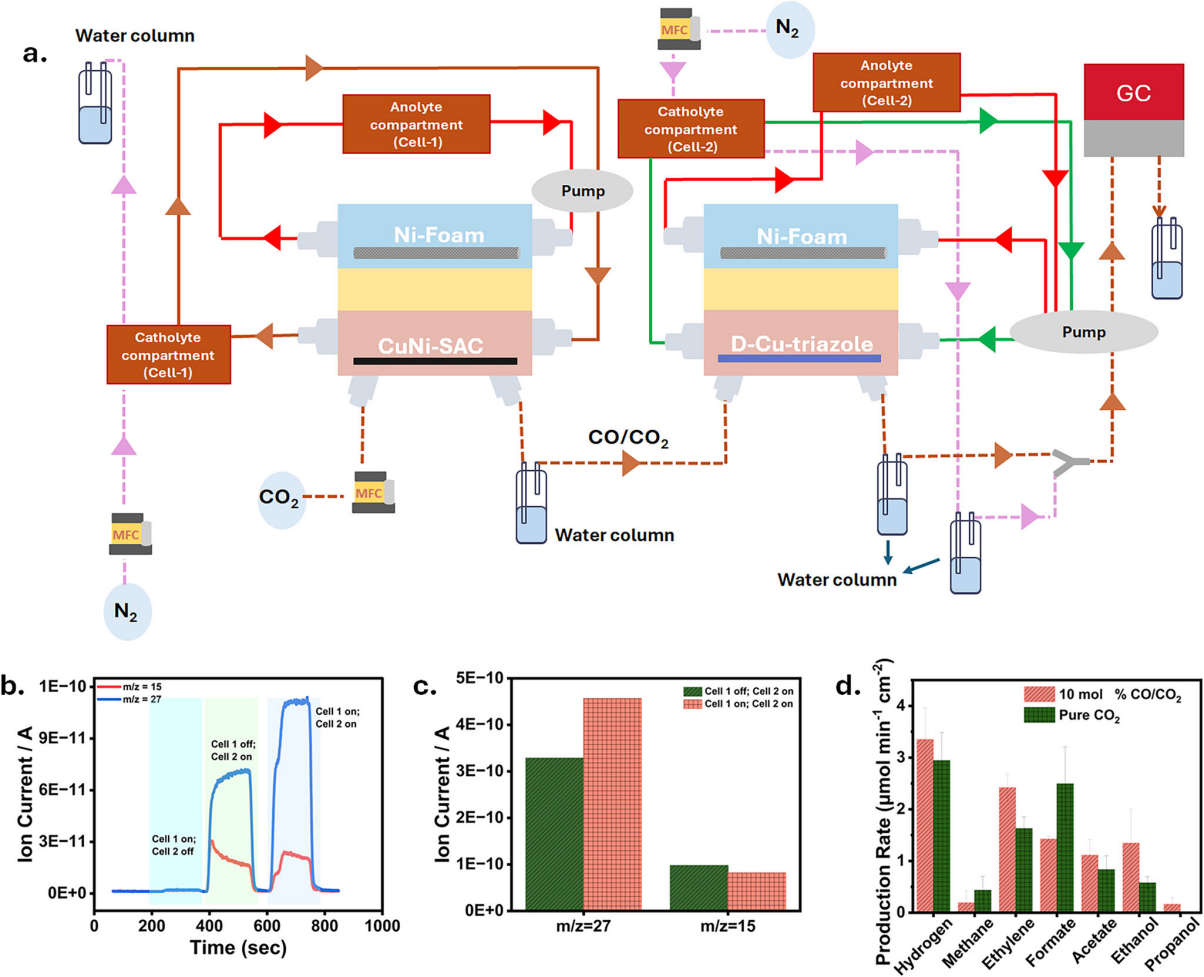
a) Electrochemical setup used for tandem cell measurements [(Cell 1‐ CO_2_ to CO at the cathode and OER at the anode) and (Cell 2‐ CO_2_/CO to C_1_ and C_2+_ products at the cathode and OER at the anode)]. b) Online electrochemical mass spectrometry (OLEMS) results for methane and ethylene, c) Signal mean value of methane and ethylene using the tandem cell configuration. d) Production rate of products in tandem cell configuration at pure CO_2_ and ∼10 mol% CO/CO_2_ concentrations.

**FIGURE 5:**
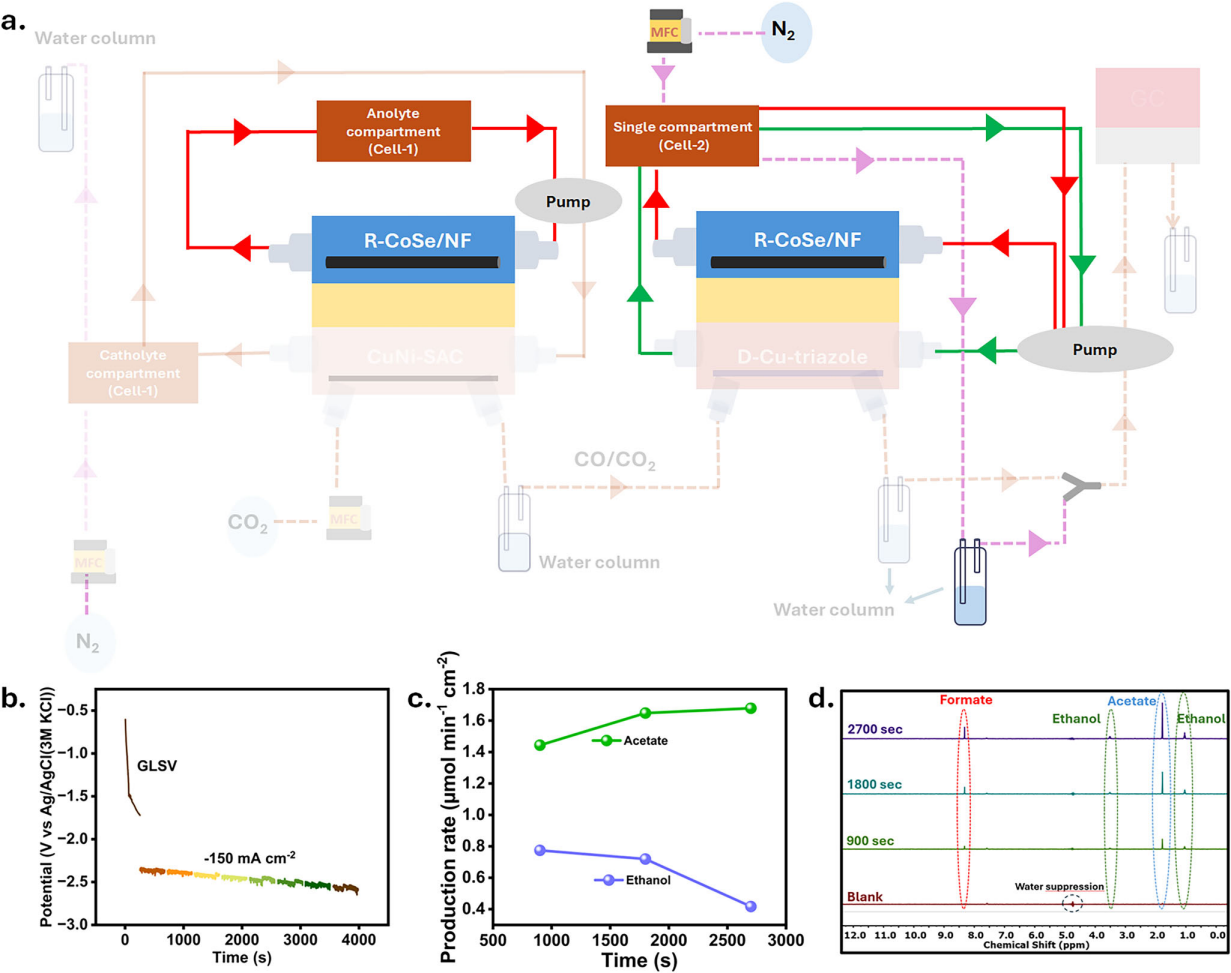
a) Modification of the electrochemical setup with respect to the one shown in Figure 4a used for coupled tandem cell measurement [(cell‐1 ‐ CO_2_ to CO at the cathode and ethanol to acetate at anode) and (cell‐2 ‐ CO_2_/CO to C_1_ and C_2+_ products at the cathode and ethanol to acetate at the anode with mixing of the catholyte with the anolyte to use the cathodically produced ethanol for additional acetate formation)]. b) Uncompensated potential for cell‐2 at ‐150 mA cm^−2^. c) Production rate for ethanol and acetate at cell‐2. d) ^1^H‐NMR spectra of electrolyte from cell‐2 recorded every 900 s.

Following the characterization of the reactions individually for the cathodes of cell‐1 (CO‐selective CuNi‐SAC catalyst) and cell‐2 (C_2+_ and oxygenates‐selective defect‐Cu‐triazole), we generated CO in cell‐1 (CO_2_‐to‐CO) and performed eCO/CO_2_RR in cell‐2 (CO/CO_2_‐to‐C_2+_), with OER as the anode reactions in both cells (Figure 4a), to comprehend selectivity modulations in cell‐2 caused by the presence of CO in the CO_2_ gas stream. We coupled the electrolyzer to an online electrochemical mass spectrometry (OLEMS) system to monitor the generated volatile products, detecting H_2_ (m/z = 2), CH_4_ (m/z = 15), and ethylene (m/z = 26). Since the eCO_2_RR selectivity is potential dependent [49], we performed the OLEMS measurements at a constant applied potential of ‐2.0 V for cell‐2. For CO‐generation cell‐1 was operated at a constant current density of ‐400 mA cm^−2^. A measurement cycle comprised switching on cell‐1 and identifying the products (primarily CO), switching off cell‐1 and turning on cell‐2, to identify the eCO_2_RR selectivity in the absence of CO in the gas stream (mainly ethylene and methane), and finally switching on both cells, to evaluate the selectivity modulation due to the presence of CO in the gas feed for cell‐2. A significant rise in the ethylene signal was observed together with a slight decrease in the methane signal, suggesting that the production rate of C_2+_ products increased in the presence of CO in the gas stream of cell‐2 (Figure 4b,c). The production rates of the liquid products were determined by means of high‐performance liquid chromatography of the catholyte and anolyte compartments of cell‐1 and cell‐2. For this, both cells were operated galvanostatically with an applied current of ‐500 mA cm^−2^ in cell‐1 in 1 M KOH (Figure S17), and ‐150 mA cm^−2^ in cell‐2 also in 1 M KOH, respectively (Figure S18). After 30 min, cell one was turned off, and the measurement was continued for an additional 30 min. In the presence of CO in the gas stream, the C_1_ production rate decreases, while the production rate of C_2+_ products like ethylene, acetate, ethanol, and propanol increases in agreement with the OLEMS measurements (Figure 4c). The production rate for methane formation decreased from 0.44 µmol min^−1^ cm^−2^ to 0.19 µmol min^−1^ cm^−2^ and for formate from 2.49 to 1.43 µmol min^−1^ cm^−2^, while the production rate for ethylene increased from 1.63 µmol min^−1^ cm^−2^ to 2.42 µmol min^−1^ cm^−2^ and for acetate from 0.83 µmol min^−1^ cm^−2^ to 1.12 µmol min^−1^ cm^−2^. The ethanol production rate also increased from 0.58 to 1.35 µmol min^−1^ cm^−2^ (Figure 4d). Furthermore, we integrated transformed CoSe/NF as anode catalyst in both cells to replace the OER with the eEOR. To maximize the production rate for acetate, we recirculated the ethanol produced in the cathode compartment of cell‐2 to the anolyte by using a single electrolyte container, as shown schematically in Figure 5a. For a better understanding of the modification of the electrolyser setup, we highlighted the modification with respect to the initial setup as shown in Figure 4a. To evaluate the ethanol to acetate conversion in both cells, we performed a long‐term measurement with cell‐1 running at ‐300 mA cm^−2^ ((CuNi‐SAC as cathode (1 M KOH) and CoSe/NF as anode (1 M KOH + 1 M EtOH)) (Figure S19), achieving a production rate for acetate of 39 µmol min^−1^ cm^−2^ (Figure S20), further confirmed by ^1^H NMR spectroscopy (Figure S21). The product formation pathways are shown in Figure S22. Cell‐2 was operated at ‐150 mA cm^−2^ with D‐Cu‐triazole as cathode and CoSe/NF as anode and a combined electrolyte (1 M KOH) as shown in Figure 5b. The acetate production rate increased from 1.44 µmol min^−1^ cm^−2^ after 15 min of electrolysis to 1.68 µmol min^−1^ cm^−2^ after 45 min (Figure 5c,d).

## 3 Conclusion

We proposed and characterized a Ni foam‐supported cobalt selenium catalyst for electrochemical ethanol oxidation to acetate. We achieved a production rate of 95.2 µmol cm^−1 ^min^−1^ using a flow‐through electrolyzer at a high current density of 1 A cm^−2^. Ex‐situ and operando techniques confirmed a structural transformation, exposing Co active sites. To expand the scope of the work, we coupled this system with electrochemical CO_2_ reduction by employing a NiCu single‐site catalyst in cell‐1 of a tandem cell system, providing CO for a defective Cu triazole catalyst on the gas diffusion cathode of cell‐2. This significantly enhanced the production rate of C_2+_ products, including acetate and ethanol. The ethanol synthesized at the cathode of cell‐2 was oxidized to acetate at the anode of cell‐2 by combining the anolyte and the catholyte, leading to a substantially enhanced acetate formation using on the one hand ethanol and on the other hand CO_2,_ probably at a later stage from fermentation processes, providing a pathway for a possibly CO_2_‐negative bioethanol to acetate conversion. Looking ahead, industrial application will require optimization of the acetate concentration in the product stream as well as downstream processing. Such engineering‐focused refinements can be a promising direction for future development.

## Conflicts of Interest

The authors declare no conflicts of interest.

## Supporting information




**Supporting File 1**: anie72056‐sup‐0001‐SuppMat.docx.

## Data Availability

The data that support the findings of this study are openly available in Zenodo at https://zenodo.org/, reference number 10.5281/zenodo.18367007

## References

[1] A. Kazmi , T. Sultana , A. Ali , A. Nijabat , G. Li , and H. Hou , “Innovations in Bioethanol Production: A Comprehensive Review of Feedstock Generations and Technology Advances,” Energy Strategy Reviews 57 (2025): 101634, 10.1016/j.esr.2024.101634.

[2] R. El‐Araby , “Biofuel Production: Exploring Renewable Energy Solutions for a Greener Future,” Biotechnol Biofuels Bioprod 17 (2024): 129.PMID: 39407282. 39407282 10.1186/s13068-024-02571-9 PMC11481588

[3] R. Samanta , A. Shekhawat , P. Sahu , and S. Barman , “Review and Perspective of Nickel and Its Derived Catalysts for Different Electrochemical Synthesis Reactions in Alkaline media for Hydrogen Production,” Energy Fuel 38 (2024): 73–104, 10.1021/acs.energyfuels.3c03758.

[4] A. Giehl , N. Klanovicz , A. F. Camargo , M. L. R. Albarello , H. Treichel , and S. L. Alves , “Ethanol and Electricity: Fueling or Fooling the Future of Road Passenger Transport?,” Energy Nexus 12 (2023): 100258, 10.1016/j.nexus.2023.100258.

[5] A. Shekhawat , R. Samanta , S. Panigrahy , and S. Barman , “Electrocatalytic Oxidation of Urea and Ethanol on Two‐dimensional Amorphous Nickel Oxide Encapsulated on N‐doped Carbon Nanosheets,” ACS Appl Energy Mater 6 (2023): 3135–3146, 10.1021/acsaem.3c00151.

[6] A. Limayem and S. C. Ricke , “Lignocellulosic Biomass for Bioethanol Production: Current Perspectives, Potential Issues and Future Prospects,” Progress in Energy and Combustion Science 38 (2012): 449–467, 10.1016/j.pecs.2012.03.002.

[7] T. Su , D. Zhao , M. Khodadadi , and C. Len , “Lignocellulosic Biomass for Bioethanol: Recent Advances, Technology Trends, and Barriers to Industrial Development,” Current Opinion in Green Sustainable Chemistry 24 (2020): 56–60, 10.1016/j.cogsc.2020.04.005.

[8] S. Jain and S. Kumar , “Advances and Challenges in Pretreatment Technologies for Bioethanol Production: A Comprehensive Review,” Sustainable Chemistry for Climate Action 5 (2024): 100053, 10.1016/j.scca.2024.100053.

[9] H. Zhang , P. Zhang , T. Wu , and H. Ruan , “Bioethanol Production Based on Saccharomyces Cerevisiae: Opportunities and Challenges,” Fermentation 9 (2023): 709, 10.3390/fermentation9080709.

[10] G. Walker and G. Stewart , “Saccharomyces Cerevisiae in the Production of Fermented Beverages,” Beverages 2 (2016): 30, 10.3390/beverages2040030.

[11] Y. Xu , L. Isom , and M. A. Hanna , “Adding Value to Carbon Dioxide From Ethanol Fermentations,” Bioresource Technology 101 (2010): 3311–3319, 10.1016/j.biortech.2010.01.006. 20110166

[12] S. Mostrou , T. Sipőcz , A. Nagl , et al., “Catalytic Oxidation of Aqueous Bioethanol: An Efficient Upgrade From Batch to Flow,” Reaction Chemistry & Engineering 3 (2018): 781–789, 10.1039/C8RE00054A.

[13] A. Sohail , W. Nunthakitgoson , S. Klinyod , et al., “Simultaneous Electrochemical Upgrading of Biomass and CO_2_ Utilization Using Fe/Ni‐derived Carbon Nanotubes Derived From CO_2_ ,” Angewandte Chemie International Edition 64 (2025): e202501404, 10.1002/anie.202501404. 40069103

[14] C. Lu , S. Yang , P. Shi , et al., “Integrated Electrochemical Biomass Oxidation and CO_2_ Reduction Over Ultra‐wide Potential Window,” Angewandte Chemie International Edition 64 (2025): e202502846, 10.1002/anie.202502846. 39989097

[15] A. Shekhawat , M. A. A. Mahbub , and W. Schuhmann , “Electrochemical CO_2_ Reduction Toward Acetate/Acetic Acid,” ChemElectroChem 13 (2026): e202500413, 10.1002/celc.202500413.

[16] Y. Zhang , W. Zhu , J. Fang , et al., “Electrochemical Converting Ethanol to Hydrogen and Acetic Acid for Large Scale Green Hydrogen Production,” Nano Research 17 (2024): 1542–1551, 10.1007/s12274-023-6023-1.

[17] S. Varhade , A. Guruji , C. Singh , et al., “Electrochemical CO_2_ Reduction: Commercial Innovations and Prospects,” ChemElectroChem 12 (2025): e202400512, 10.1002/celc.202400512.

[18] B. S. Crandall , B. H. Ko , S. Overa , et al., “Kilowatt‐scale Tandem CO_2_ Electrolysis for Enhanced Acetate and Ethylene Production,” Nature Chemical Engineering 1 (2024): 421–429, 10.1038/s44286-024-00076-8.

[19] B. K. Manna , R. Samanta , and S. Barman , “Porous Cobalt–Nickel Binary Oxide Nanosheets for Electrochemical Glycerol Oxidation,” ACS Appl Energy Mater 7 (2024): 11787–11798, 10.1021/acsaem.4c01995.

[20] M. Braun , M. Chatwani , P. Kumar , et al., “Cobalt Nickel Boride as Electrocatalyst for the Oxidation of Alcohols in Alkaline media,” Journal of Physics Energy 5 (2023): 024005, 10.1088/2515-7655/acbb2a.

[21] D. Chen , Z. Zhao , G. Chen , et al., “Metal Selenides for Energy Storage and Conversion: A Comprehensive Review,” Coordination Chemistry Reviews 479 (2023): 214984, 10.1016/j.ccr.2022.214984.

[22] E. Gebhardt and W. Köster , “System Platin‐Kobalt mit Besonderer Berücksichtigung der Phase CoPt,” International Journal of Materials Research 32 (1940): 253–261, 10.1515/ijmr-1940-320801.

[23] C. H. Griffiths and H. Sang , “Extra Reflections in Electron Diffraction Patterns From Polycrystalline and Single Crystal Selenium Films,” Materials Research Bulletin 2 (1967): 515–519, 10.1016/0025-5408(67)90026-8.

[24] L. Huey‐Lin and P. Duwez , “Solid Solutions of Rhodium With Copper and Nickel,” Journal of Less‐Common Metals 6 (1964): 248–249, 10.1016/0022-5088(64)90108-0.

[25] M. C. Biesinger , B. P. Payne , A. P. Grosvenor , L. W. Lau , A. R. Gerson , and R. S. Smart , “Resolving Surface Chemical States in XPS Analysis of First Row Transition Metals, Oxides and Hydroxides: Cr, Mn, Fe, Co and Ni,” Applied Surface Science 257 (2011): 2717–2730, 10.1016/j.apsusc.2010.10.051.

[26] J. F. Moulder , W. F. Stickle , P. E. Sobol , and K. D. Bomben , Handbook of X‐ray Photoelectron Spectroscopy, Physical Electronics Division (Perkin‐Elmer Corporation, 1992).

[27] X. Xiao , Y. Wang , B. Cui , X. Zhang , D. Zhang , and X. Xu , “Preparation of MoS_2_ Nanoflowers With Rich Active Sites as an Efficient Adsorbent for Aqueous Organic Dyes,” New Journal of Chemistry 44 (2020): 4558–4567, 10.1039/D0NJ00129E.

[28] J. Zhang , P. Leung , F. Qiao , et al., “Balancing the Electron Conduction and Mass Transfer: Effect of Nickel Foam Thickness on the Performance of an Alkaline Direct Ethanol Fuel Cell (ADEFC) With 3D Porous Anode,” International Journal of Hydrogen Energy 45 (2020): 19801–19812, 10.1016/j.ijhydene.2020.05.119.

[29] G. Li , L. Anderson , Y. Chen , M. Pan , and P.‐Y. Abel Chuang , “New Insights Into Evaluating Catalyst Activity and Stability for Oxygen Evolution Reactions in Alkaline media,” Sustain Energy Fuels 2 (2018): 237–251, 10.1039/C7SE00337D.

[30] B. Dasgupta , S. Yao , I. Mondal , et al., “A Knowledge‐based Molecular Single‐Source Precursor Approach to Nickel Chalcogenide Precatalysts for Electrocatalytic Water, Alcohol, and Aldehyde Oxidations,” ACS nano 18 (2024): 33964–33976, 10.1021/acsnano.4c08058. 39626115 PMC11656844

[31] Q. Wen , Y. Lin , Y. Yang , et al., “In Situ Chalcogen Leaching Manipulates Reactant Interface Toward Efficient Amine Electrooxidation,” ACS nano 16 (2022): 9572–9582, 10.1021/acsnano.2c02838. 35679123

[32] B. Sun , W. Zhong , X. Ai , C. Zhang , F.‐M. Li , and Y. Chen , “Engineering Low‐coordination Atoms on RhPt Bimetallene for 12‐electron Ethanol Electrooxidation,” Energy & Environmental Science 17 (2024): 2219–2227, 10.1039/D3EE04023B.

[33] R. L. Frost and E. C. Keeffe , “Raman Spectroscopic Study of the Selenite Mineral: Ahlfeldite, NiSeO_3_ 2 H_2_O,” Journal of Raman Specroscopy 40 (2009): 509–512, 10.1002/jrs.2075.

[34] M. Sun , D. S. R. Rocabado , J. Cheng , et al., “In Situ Observation of Post‐CO Intermediates to Decode C‐C Coupling Pathways in CO_2_ Electroreduction,” Angewandte Chemie International Edition 64 (2025): e202502740, 10.1002/anie.202502740. 40338611 PMC12281082

[35] F. Shao , Z. Xia , F. You , et al., “Surface Water as an Initial Proton Source for the Electrochemical CO Reduction Reaction on Copper Surfaces,” Angewandte Chemie International Edition 62 (2023): e202214210, 10.1002/anie.202214210. 36369647

[36] J. F. Gomes , K. Bergamaski , M. F. Pinto , and P. B. Miranda , “Reaction Intermediates of Ethanol Electro‐oxidation on Platinum Investigated by SFG Spectroscopy,” Journal of Catalysis 302 (2013): 67–82, 10.1016/j.jcat.2013.02.024.

[37] J. Li , Y. Kuang , X. Zhang , et al., “Electrochemical Acetate Production From High‐pressure Gaseous and Liquid CO_2_ ,” Nature Catalysis 6 (2023): 1151–1163, 10.1038/s41929-023-01046-8.

[38] J. G. Chen , J. E. Crowell , and J. T. Yates , “An EELS and TPD Study of the Adsorption and Decomposition of Acetic Acid on the Al(111) Surface,” Surface Science 172 (1986): 733–753, 10.1016/0039-6028(86)90509-1.

[39] A. Shekhawat , D. Das , R. Zerdoumi , et al., “Defect‐induced Selectivity Modulation Using Copper Triazole Molecular Frameworks for Electrochemical CO_2_ Reduction,” Advanced Functional Materials 35 (2025): 2506172, 10.1002/adfm.202506172.

[40] L. Yang , X. Lv , C. Peng , et al., “Promoting CO_2_ Electroreduction to Acetate by an Amine‐terminal, Dendrimer‐functionalized Cu Catalyst,” ACS Cent Sci 9 (2023): 1905–1912, 10.1021/acscentsci.3c00826. 37901173 PMC10604016

[41] P. Wei , D. Gao , T. Liu , et al., “Coverage‐driven Selectivity Switch From Ethylene to Acetate in High‐rate CO_2_/CO Electrolysis,” Nature Nanotechnology 18 (2023): 299–306, 10.1038/s41565-022-01286-y. 36635334

[42] T. Möller , M. Filippi , S. Brückner , W. Ju , and P. Strasser , “A CO_2_ Electrolyzer Tandem Cell System for CO_2_‐CO co‐feed Valorization in a Ni‐N‐C/Cu‐catalyzed Reaction Cascade,” Nature Communications 14 (2023): 5680. 10.1038/s41467-023-41278-7 PMC10502113 37709744

[43] J. Weidner , C. N. Tchassem , D. Das , et al., “Al‐rich Cu/CuO_x_ Catalyst in a CO_2_‐reduction Tandem Electrolyzer With CO‐enriched Gas Feed for Enhanced C_2+_‐products Selectivity,” ChemElectroChem 12 (2025): e202500664, 10.1002/celc.202400664.

[44] G. Lu , X. Wang , J. Timoshenko , et al., “A 3D Macroporous Carbon NiCu Single‐atom Catalyst for High Current Density CO_2_ Electroreduction,” Advanced Functional Materials 35 (2025): 2419075, 10.1002/adfm.202419075.

[45] Y. C. Tan , K. B. Lee , H. Song , and J. Oh , “Modulating Local CO_2_ Concentration as a General Strategy for Enhancing C−C Coupling in CO_2_ Electroreduction,” Joule 4 (2020): 1104–1120, 10.1016/j.joule.2020.03.013.

[46] W. Plischka , M. Heßelmann , M. Wessling , and R. Keller , “Modeling Different Wetting States in Gas Diffusion Electrodes for CO_2_ Electrolysis,” Electrochimica Acta 537 (2025): 146699, 10.1016/j.electacta.2025.146699.

[47] H. Kim , W. Jang , J. H. Lee , et al., “Energy‐Efficient Dual Formate Electrosynthesis via Coupled Formaldehyde Oxidation and CO2 Reduction at Ultra‐Low Cell Voltage,” Angewandte Chemie International Edition 64 (2025): e202516232, 10.1002/anie.202516232. 41031702

[48] Z. Zheng , X. Zheng , L. Wang , et al., “Harnessing Electrocatalytic Coupling of Carbon Dioxide and Methanol for High‐Efficiency Formic Acid Production,” Angewandte Chemie International Edition 64 (2025): e202512078, 10.1002/anie.202512078. 40913385 PMC12535387

[49] B. Chang , H. Pang , F. Raziq , et al., “Electrochemical Reduction of Carbon Dioxide to Multicarbon (C_2+_) Products: Challenges and Perspectives,” Energy & Environmental Science 16 (2023): 4714–4758, 10.1039/D3EE00964E.

