# Supplementary material for: Coupling Electrochemical CO2 Reduction With Ethanol Oxidation for Acetate Production in a Dual‐Electrolyzer System
Source: Angew Chem Int Ed Engl. 2026 Apr 2;65(20):e9339732. doi: 10.1002/anie.9339732 (PMC13159404; doi:10.1002/anie.9339732)
Supplement: Supplementary file 1 — Supporting File 1: anie72056‐sup‐0001‐SuppMat.docx. [file ANIE-65-e9339732-s001.docx]

Supporting Information

Coupling Electrochemical CO_2_ Reduction with Ethanol Oxidation for Acetate Production in a Dual-Electrolyzer System

Anirudha Shekhawat, ^[a]^ Shubhadeep Chandra, ^[a]^ Ridha Zerdoumi, ^[a]^ Bashir Eid, ^[a]^ Muhammad Adib Abdillah Mahbub, ^[a]^ Wolfgang Schuhmann*^[a]^

**Experimental Sections**

**Materials:** All chemicals were obtained commercially and used without further purification. Ethylene­diamine, cobalt (II) nitrate hexahydrate, nickel (II) nitrate hexahydrate, copper(II) nitrate trihydrate, 1H-1,2,3-triazole, selenium nanopowder, tetraethyl orthosilicate, polyvinylpyrrolidone (PVP) K30 (Mw ∼ 40,000), 37 wt.% formaldehyde, Poly(tetrafluroroethylene) powder 1µm particle size and sodium hydroxide were from Sigma Aldrich. Ammonia 25%, ethanol, methanol, isopropyl alcohol, and N,N-diethylformamide were from VWR Chemicals. KOH was from Fisher Scientific. Deionised water was from a Millipore purification system (18.2 MΩ). A Chelex® resin column purified the KOH electrolyte before use.

**Synthesis:**

**Cobalt selenium-nickel foam (CoSe/NF)**

Nickel foam (2.5 cm × 3.5 cm) was soaked in 50 vol% HCl solution to remove surface contaminations, followed by cleaning with deionized water and ethanol. Cobalt (II) nitrate hexahydrate (2.90 g) and Se powder (3.15 g) were transferred into the Teflon container with a solution of 36 mL water and 24 mL ethylenediamine. The nickel foam was immersed into the solution and was sonicated for 30 min. The Teflon container was sealed in a stainless-steel autoclave and kept for 4 h at 140 °C. After cooling down, the cobalt selenium modified nickel foam was taken out, rinsed 3 times with water, and dried at 70 °C in an oven.

**Defective copper-1H-1,2,3-triazole MOF (D-Cu-triazole)**

D-Cu-triazole was prepared according to our previously reported procedure.^[1]^ 242 mg of copper (II) nitrate trihydrate were dissolved in 10 mL N,N-diethylformamide in a 22 mL screw vial and stirred for 5 min using a vortex mixer. After that, 207 mg of 1H-1,2,3-triazole was added. This mixture was kept in an oven at 100 °C for 18 h and washed thrice with N,N-diethylformamide. After that, it was kept in methanol for three days and washed 3 times with methanol. The D-Cu-triazole MOF was obtained after drying in vacuo and then in an oven at 70 °C.

**Copper nickel-single atom catalyst**

The CuNi-SACs were synthesised according to a previous report with minor modifications.^[2]^ Initially, a mesoporous SiO_2_ template was prepared. 5 ml of tetraethyl orthosilicate (TEOS) was added to 50 ml of ethanol and heated to 40 °C (Solution A). Then a solution containing 25 ml ethanol, 5 ml aqueous ammonia, and 10 ml DI water was made (Solution B). Solution B was added to solution A and stirred at 40 °C for 2.5 h. A mixture containing 10 mL TEOS, 5 g polyvinylpyrrolidone (PVP) K30 (Mw ≈ 40,000), and 10 mL ethanol was added to the silica sol under vigorous stirring at 40 °C for 2 h. After cooling down, the solution was centrifuged, and the obtained sample was dried at 70 °C and further calcined at 700 °C for 6 h.

After that, 0.75 g of the SiO_2_ template and 1.5 g dicyandiamide were mixed in 10 ml DI water and stirred for 1.5 h. 0.11 mmol copper (II) nitrate trihydrate, 0.11 mmol nickel (II) nitrate hexahydrate, and 1.6 ml of formaldehyde were added and heated to 60 °C for 4 h. After that, the solvent was evaporated, and the obtained solid was heated to 600 °C for 2 h in an Ar atmosphere. The product was further immersed in 5 M NaOH for 15 h and then washed with DI water. The product was further treated in 1 M HCl, washed with water, and dried in an oven at 70 °C to obtain the catalyst.

**Characterization**

X-ray diffraction (XRD) data were obtained using a Bruker D8 Discover X-ray diffractometer equipped with a Cu K_α_ radiation source (λ = 1.5418 Å) in the range of 5° to 70°. The CoSe modified nickel foam and powder sample for CuNi-SACs were placed on a low-background silicon wafer in a PMMA holder. Field-emission scanning electron micrographs (FE-SEM) were recorded using a Quanta 3D ESEM operated at 30 kV. For FE-SEM measurements, CoSe modified nickel foam was kept on a carbon tape on the sample holder. The CuNi-SACs powder was adsorbed on a carbon tape. Transmission electron micrographs (TEM), high-resolution TEM, and energy dispersive spectroscopy (EDS) elemental mapping were obtained using a JEOL microscope (JEM-2800) with a Schottky-type emission source working at 200 kV. CoSe modified nickel foam was inserted in isopropanol and sonicated for 15 min. After that, 10 µL of the material was drop-cast on a carbon-supported gold TEM grid. The near-surface composition of the samples was investigated using X-ray photoelectron spectroscopy (XPS). An AXIS Nova spectrometer (Kratos Analytical) equipped with a monochromatic Al K_α_ X-ray source (1487 eV, 15 mA emission current) was used. The pressure in the sample analysis chamber was around 10^-8^ Torr. Photoelectrons were collected in the fixed transmission mode while charge neutralization was applied using an electron flood gun. A pass energy of 20 eV was used to acquire survey and high-resolution spectra of the Co 3p, Se 3d, C 1s, and O 1s regions. The binding energies of the core-level spectra were calibrated based on the C 1*s* signal at 284.8 eV. Data processing and peak fitting of different components in the XPS spectra were conducted using the ESCApe software package (Kratos). Peak fitting was performed with a combination of Gaussian and Lorentzian line shapes, and the Shirley algorithm was used for background subtraction. Thermogravimetric analysis was performed using a Cahn TG 2131 thermobalance and a Thermostar MS (Pfeiffer Vacuum).

**Electrochemical Measurements**

**a. Electrochemical ethanol oxidation (eEOR)**

The working electrode (anode) was CoSe/NF, and the counter electrode (cathode) was nickel foam connected with Ni wires. A double-junction Ag|AgCl|KCl (3 M) was the reference electrode with 1 M KOH in the outer compartment. A cation exchange membrane (CEM) was used (FKL-PK-130, Fumasep) to separate the anodic and cathodic compartments. The preconditioning steps were performed in 1M KOH using 5 CV from 0 V to 0.6 V vs AgCl (3M KCl) followed by 5 consecutive LSV. The electrolyte for the anodic compartment was (1 M KOH + 1M EtOH), and for the cathodic compartment was (1 M KOH) with 21.17 ml volume of electrolyte in both the containers.

**b. Electrochemical CO_2_ reduction reaction (eCO_2_RR)**

The working electrode (cathode) was the D-Cu-triazole molecular catalyst for C_2+_ products generation and CuNi-SAC catalyst for CO production. The counter electrode (anode) was Ni foam connected to a nickel wire. A double-junction Ag|AgCl|KCl (3 M) was the reference electrode with 1 M KOH in the outer compartment with 20 ml volume. An anion exchange membrane (AEM) was used (FAA-PK-130, Fumasep) to separate the anodic and cathodic compartments. D-Cu-triazole-modified GDEs were prepared using airbrush-assisted reduced pressure filtration onto the gas diffusion layer (GDL; 18 mm diameter; H23C6, Freudenberg) with a loading of about 1.39 mg cm^-2^. The ink slurry was prepared by dispersing 10 mg of the catalyst material, 5 mg PTFE, and 10 µL Nafion in 5 mL isopropanol by sonication with a tip sonicator for 20 min at 20% amplitude. The ink slurry for CuNi-SAC was prepared using 2 mg catalysts and 1 mg PTFE in 2 ml isopropanol and 10 µL Nafion but drop-coated using the reduced-pressure filtration system. The catalyst loading for both catalysts was about 1.3 mg cm^-2^. The obtained GDEs were dried in vacuum and in an oven at 70 °C.

**c. Electrochemical coupled electrolysis measurement (Cell-1):**

The CuNi-SAC cathode was connected as working electrode, and the CoSe/NF anode as counter electrode. The reference electrode was a double-junction Ag|AgCl|KCl (3 M) filled with 1 M KOH in the outer compartment. A cation exchange membrane (CEM) was used (FKL-PK-130, Fumasep) to sepa­rate the anode and cathode compartments. Electrolyte for the anode compartment was 1 M KOH + 1 M EtOH (21.17 ml), and the cathode compartment was 1 M KOH (20 ml).

**d. Electrochemical tandem cell measurement (Cell-1 and Cell-2):**

The working electrode for cell-1 was CuNi-SAC, and for cell-2 a D-Cu-triazole-based GDE. The counter electrode was Ni foam connected to a Ni wire. The reference electrode was a double-junction Ag|AgCl|KCl (3 M) filled with 1 M KOH in the outer compartment. A cation exchange membrane (CEM) was used (FKL-PK-130, Fumasep) to separate the anode and cathode compartments for cell-1, and an AEM (FAA-PK-130, Fumasep) was used for cell-2.

**e. Electrochemical tandem cell with coupled electrolysis measurement (Cell-1 and Cell-2):**

The working electrode (cathode) for cell-1 was CuNi-SAC and for the cathode of cell-2 a D-Cu-triazole based GDE. The counter electrode was CoSe/NF (anode) for both cell-1 and for cell-2. The reference electrode was a double-junction Ag|AgCl|KCl (3 M) filled with 1 M KOH in the outer compartment. A cation exchange membrane (CEM) was used (FKL-PK-130, Fumasep) to separate the anode and cathode compartments for cell-1, and an AEM (FAA-PK-130, Fumasep) was used for cell-2. For cell-1, the electrolyte for the anode compartment was 1 M KOH + 1 M EtOH, and for the cathode compartment was 1 M KOH. However, for cell-2, with a volume of 30 ml (1 M KOH), the electrolyte was recirculated using a single chamber to both the anode and cathode. As a result, the in-situ formed ethanol during the eCO_2_RR at the cathode was additionally converted to acetate at the CoSe/NF anode.

All electrochemical measurements were carried out using an Autolab potentiostat (PGSTAT302N). The reference electrode potential was regularly measured against a commercial reference electrode. The reference electrode was positioned at a fixed distance from the working electrode surface. The geo­metric area of the catalyst-loaded electrodes was 0.95 cm^2^, and the geometric area was used for FE and PR calculations. The catholyte reservoir was constantly purged with 20 mL min^-1^ N_2_ while 20 mL min^-1^ of pure CO_2_ (commercial CO_2_ cylinders, AIR LIQUIDE Deutschland GmbH) was applied to the backside of the GDE unless stated otherwise. For eEOR measurements, the anolyte reservoir was constantly purged with 20 mL min^-1^ N_2_. All measurements were performed using chronopotentiometry, and the potentials were converted to the reversible hydrogen electrode (RHE) with iR correction using equation 1.

V_RHE_ = [V _Ag|AgCl|3 M KCl_ + 0.210 + (0.059*pH)] - iR ( Equation 1)

**Operando Raman measurements**

Operando Raman spectroscopy was performed with a Lab−RAM HR Raman microscopy system (Horiba Jobin-Yvon, HR550) equipped with a water immersion objective (Olympus LUMFL, 60x, numerical aperture 1.10), a monochromator (1200 grooves/mm grating), and a Synapse CCD detector. A 532 nm laser was used as the excitation source. To protect the objective lens, it was covered with a transparent Teflon film, and the measurements were performed in 1 M KOH + 1 M EtOH solution using a three-electrode set-up with a Ni-mesh as CE and an Ag|AgCl|KCl (3 M) as RE. An in-house 3D-printed Raman flow cell was used for the operando measurements. CoSe-modified NF was used as the WE. A Gamry Interface 5000E potentiostat was used for the electrochemical measurements.

**OLEMS measurements**

For OLEMS, the signals of hydrogen with mass-to-charge ratio (m/z = 2), methane (m/z = 15), ethylene (m/z = 27), carbon monoxide (m/z = 28) and carbon dioxide (m/z = 44) were recorded using a GAM 400 mass spectrometer with a secondary electron multiplier (SEM) voltage 1400 V and an emission current of 1 mA. The flow cell was connected to the MS using the gas inlet. The length of the capillary and tubing caused a signal delay of about 120 s.

**Product analysis**

Gaseous products were analysed by gas chromatography (GC, SRI instrument) with a thermal conductivity detector (TCD) to quantify H_2_ and a flame ionisation detector methanizer (FID meth) to quantify CO. The carrier gas was N_2,_ and the column temperature was 90 °C. During the measurement, the gaseous products were injected from the catholyte headspace as the top injection and the products from the CO_2_ stream (backside of GDE) as the bottom injection. The liquid product was collected from the catholyte reservoir at the end of each applied current density. The Faradaic efficiencies (FEs) were calculated for the gaseous products using equation 2.

$FE_{a}=\frac{x_{a}z_{a}fF}{V_{m}I_{t}}$ X 100 % (Equation 2)

Where FE is the Faradaic efficiency and the index “a” is related to a specific product, $x_{a}$ is the concentration of the product in vol% (conversion of ppm * 10^-6^), $z_{a}$ is the electron transfer number, $f$ is the gas flow in L s^-1^, $F$ is the Faraday constant of 96485 C mol^-1^, $V_{m}$ is the molar volume of an ideal gas at 25 °C of 24.5 L mol^-1^, and $I_{t}$ is the current in A. The product concentration in ppm was detected using Peak Simple, and the values were then used to calculate the Faradaic efficiency using Equation 2. The liquid products were analysed by high-performance liquid chromatography (HPLC, Dionex ICS-5000, ThermoFisher) with an ion-exclusion column Aminex HPX-87H (BioRad), a diode array detector, and a refractive index (RI) detector (RefractoMax520). Formate was analysed using the RI detector. The eluent was 4 mM H_2_SO_4_. The eluent flow rate was 0.6 mL min^-1^, and the column temperature was 70 °C. The sample was prepared by mixing 440 µL of collected liquid product with 110 µL of 2.5 M H_2_SO_4_ to acidify the sample. The liquid products were detected according to their specific retention times after calibration. The concentration of the products in mmol L^-1^ was provided by the software Chromelon Chromatography Studio. The value was used to determine the Faradaic efficiency for liquid products using equation 3.

$FE_{b}=\frac{n_{b}z_{b}F}{I_{t}t}$ X 100 % (Equation 3)

$n_{b}$ Is the calculated mol produced of product “b”, $z_{b}$ is the electron transfer number, and $t$ is the measurement duration.

NMR samples were prepared by mixing 400 µl of electrolyte sample with 100 µl of D_2_O. The ¹H probe was tuned and locked on the D_2_O solvent, with gradient shimming and auto-gain applied. Water peak suppression was performed, and 16 scans were collected per sample with a 6 s relaxation delay.


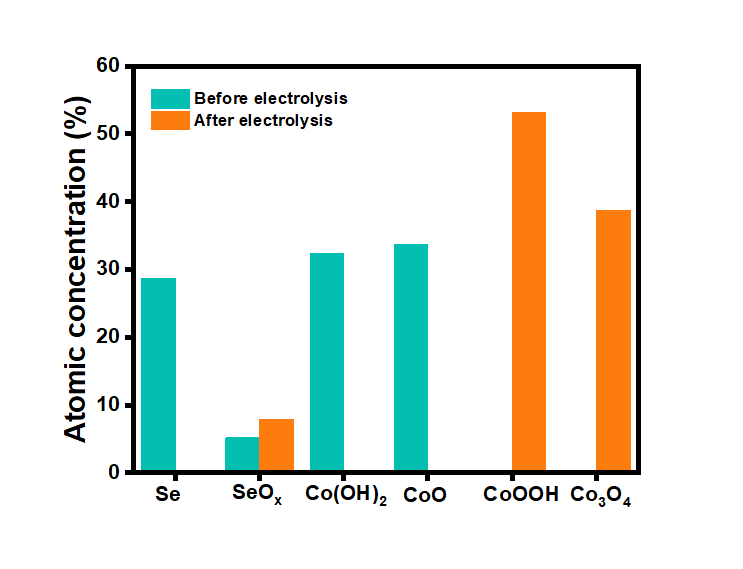


**Figure S1.** The atomic ratio of the different species before and after electrolysis of CoSe/NF catalysts was characterized through XPS measurements.


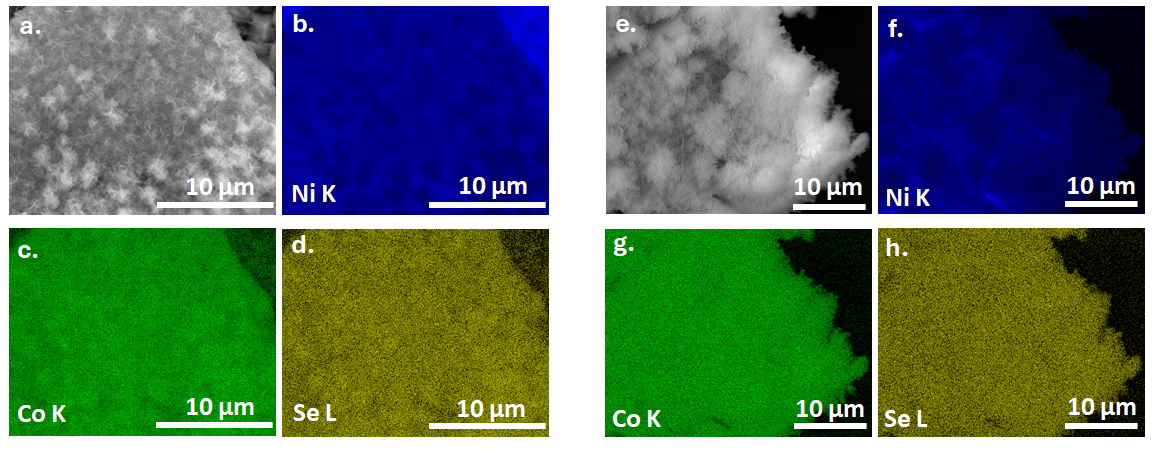
**Figure S2.** SEM-EDX images of the CoSe/NF catalyst.


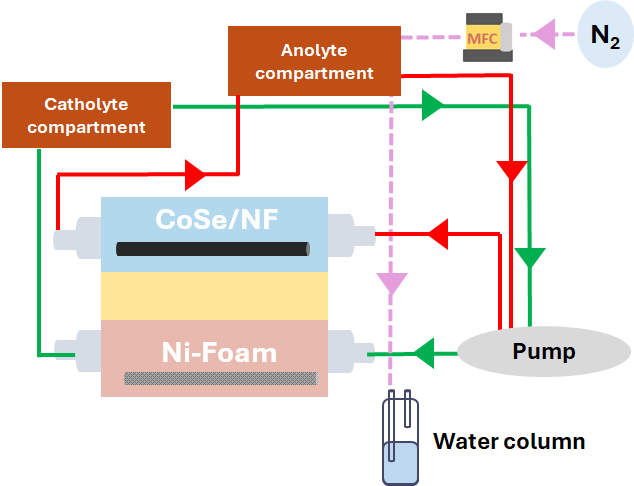


**Figure S3.** Schematic representation of anodic half-cell setup for eEOR.


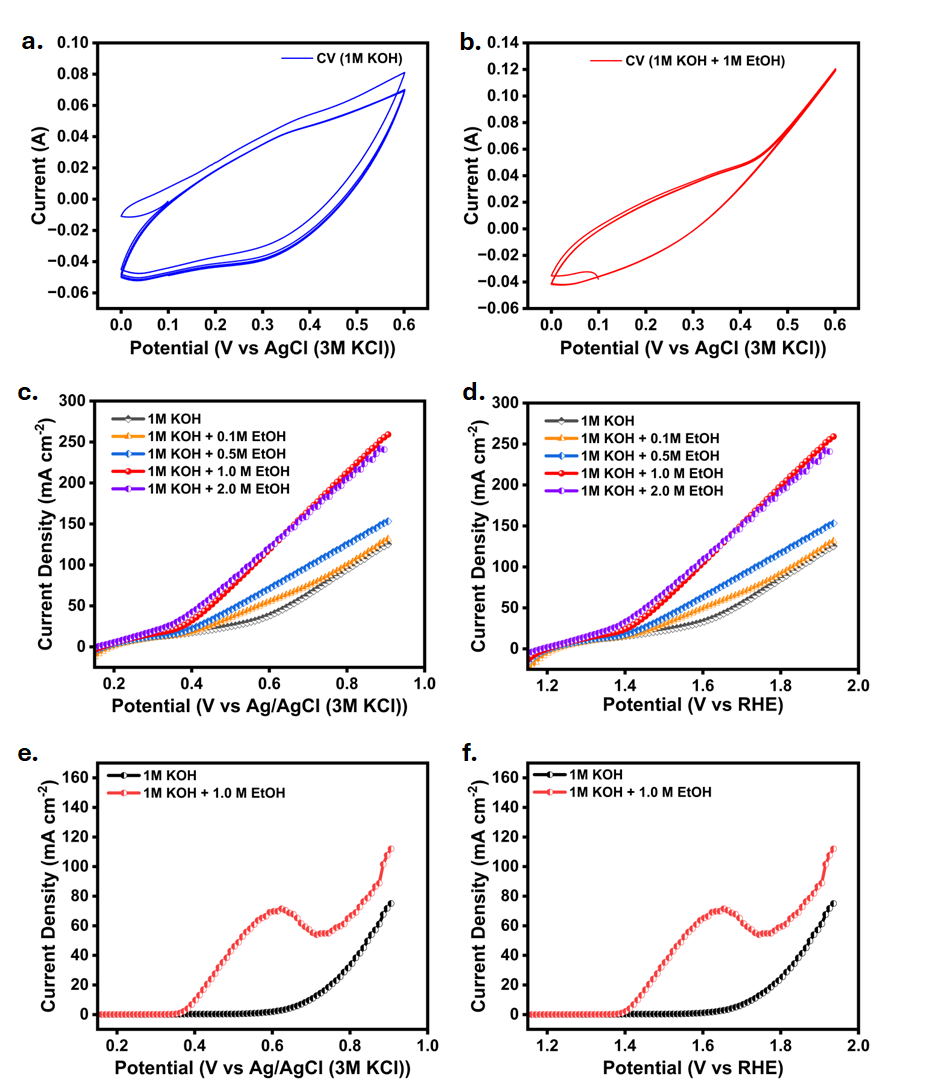


**Figure S4.** CV scans for preconditioning in (a) OER (1M KOH) and (b) EOR (1M KOH + 1M EtOH) conditions. LSVs of CoSe/NF at different concentrations of ethanol (c) with V vs Ag/AgCl (3M KCl) and (d) with V vs RHE. LSVs of bare NF in 1 M KOH + 1 M EtOH concentration with (e) V vs Ag/AgCl (3M KCl) and (f) V vs RHE.


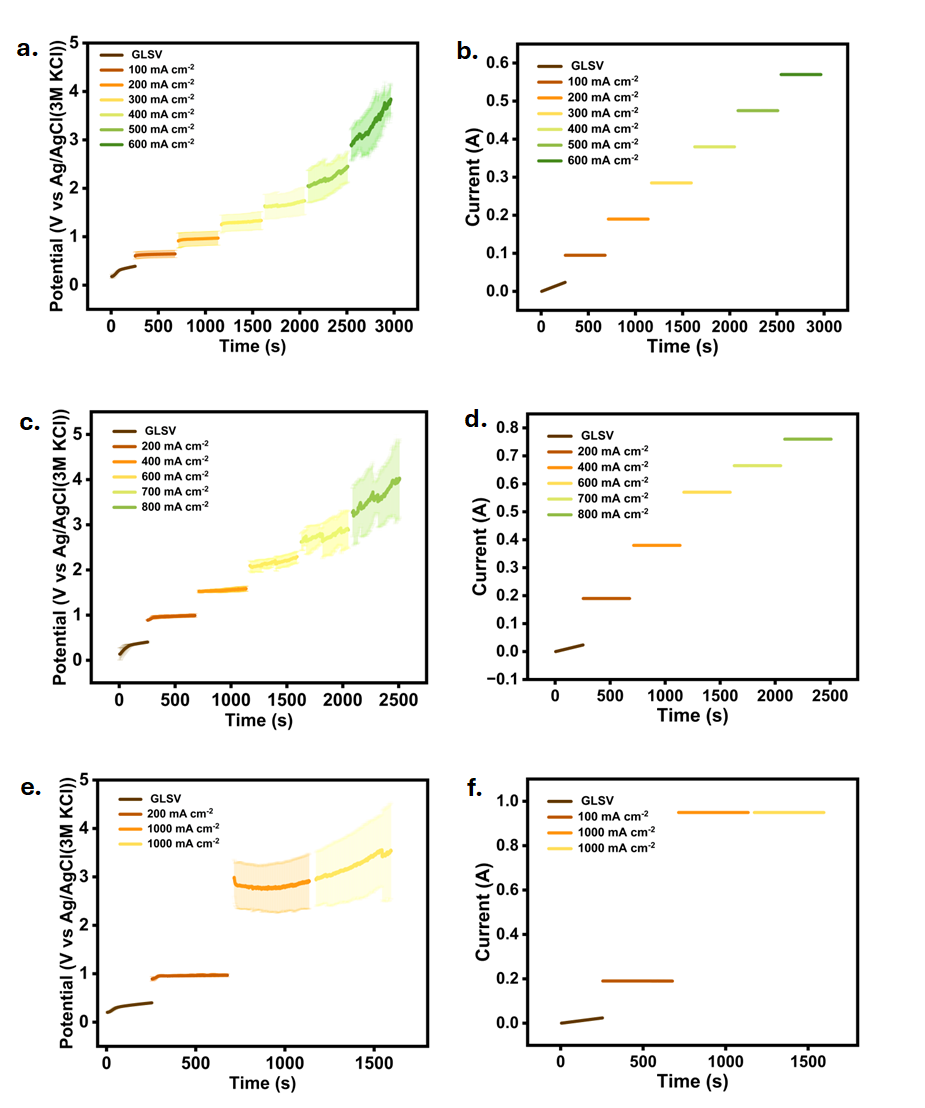
**Figure S5.** Uncompensated potential during chronopotentiometry for CoSe/NF electrodes using the same electrode in 1 M KOH + 1 M EtOH. (a,b) At different current densities, (c,d) after refreshing the electrolyte, (e,f) after refreshing the electrolyte at high current densities.


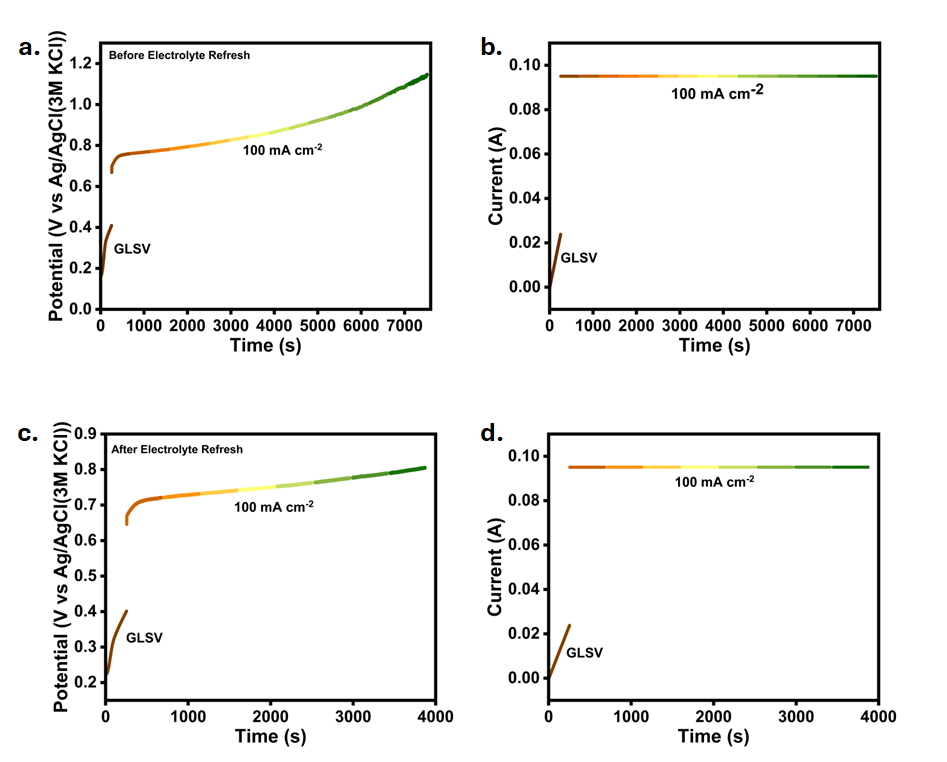
**Figure S6.** (a) Uncompensated potential during long-term chronopotentiometry at 100 mA cm^-2^ and (b) applied current for CoSe/NF (c,d) after electrolyte refreshing.


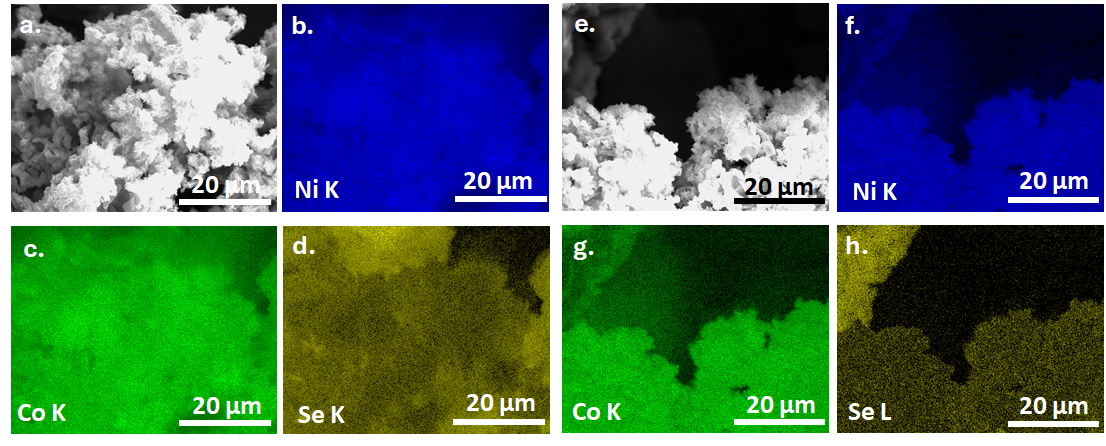


**Figure S7.** SEM-EDX images of the CoSe/NF catalyst after electrolysis.


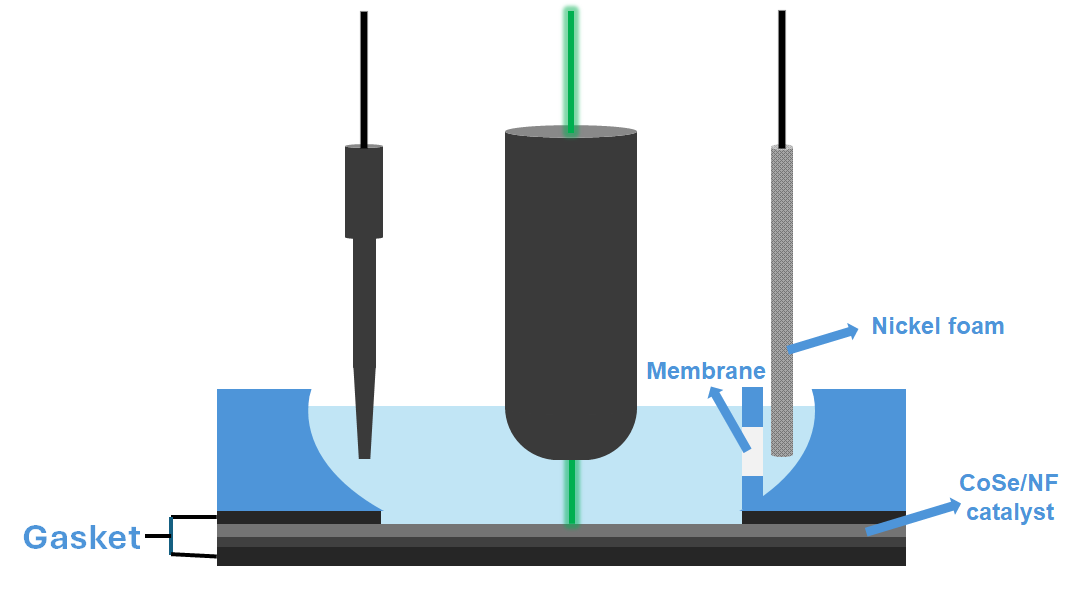
**Figure S8.** Schematic setup for the operando Raman measurements.


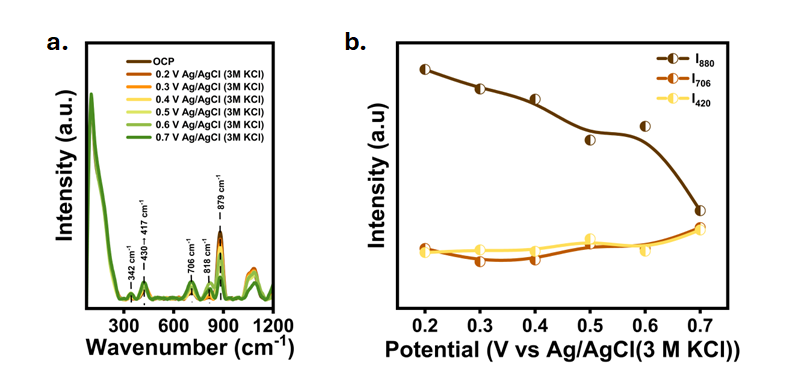


**Figure S9**. (a) Operando Raman measurement. (b) Normalized Raman spectra for quantitative analysis over increasing anodic potentials.


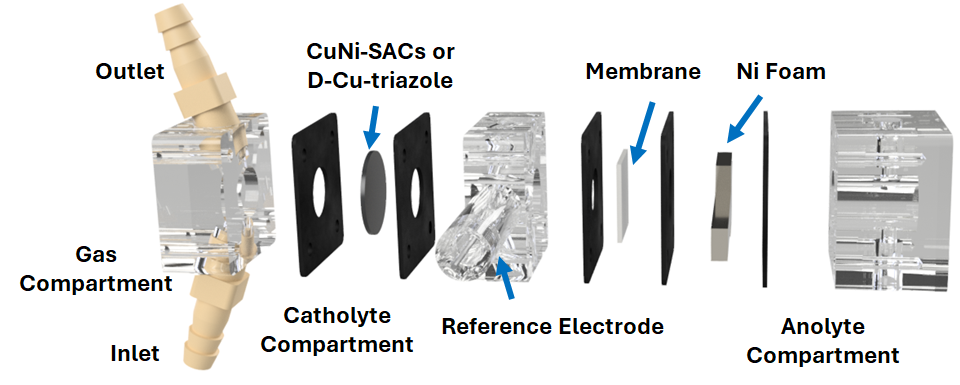


**Figure S10.** Schematic for the flow cell electrolyser used for eCO_2_RR measurements.


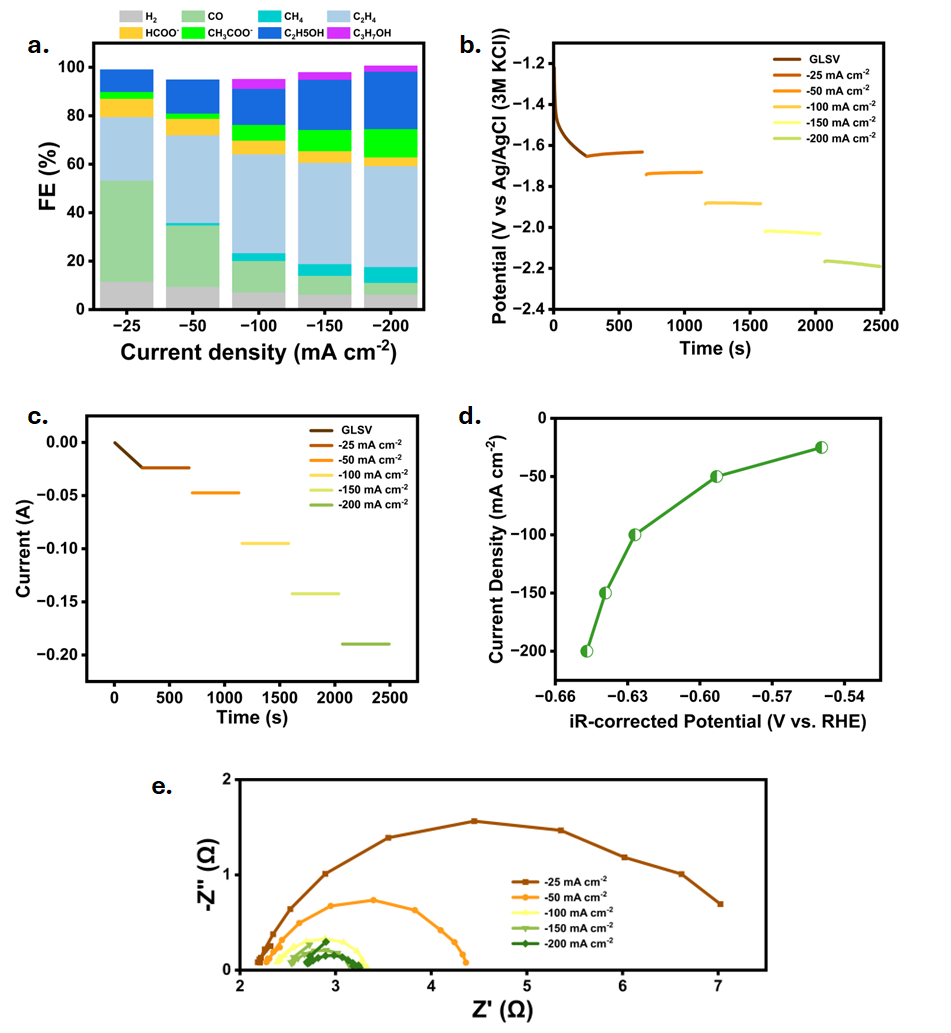


**Figure S11.** (a) FE, (b) uncompensated potential, (c) applied currents, (d) iR corrected potentials, (e) GEIS measurements for D-Cu-triazole.


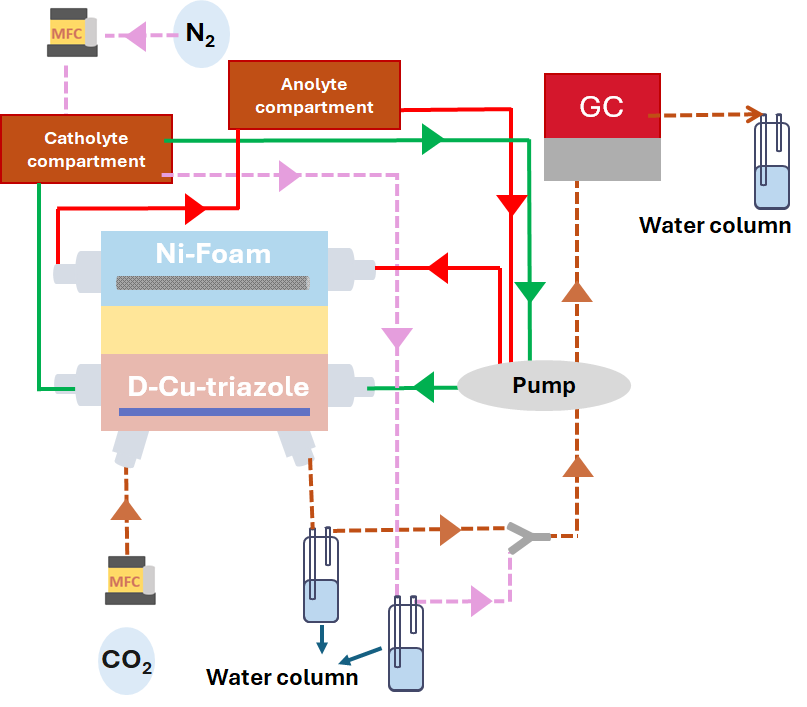


**Figure S12.** Schematic representation of the cathodic half-cell setup for eCO_2_RR.


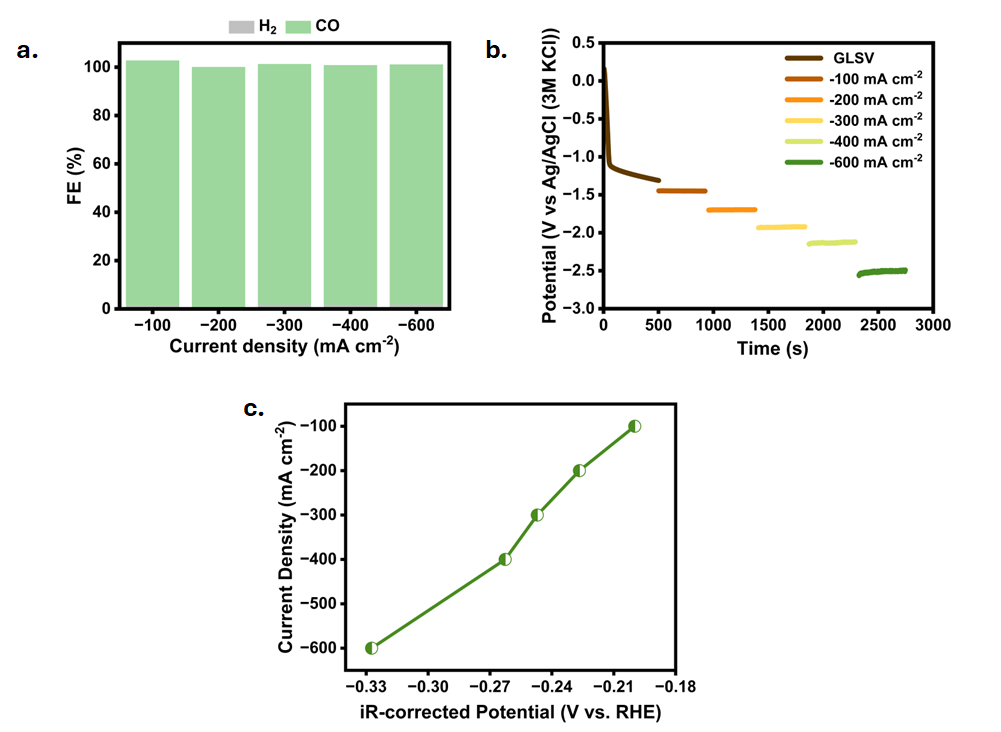


**Figure S13.** (a) FE, (b) uncompensated potential, (c) iR corrected potentials for the CuNi-SAC catalyst modified electrode at a CO_2_ flux of 20 ml min^-1^.


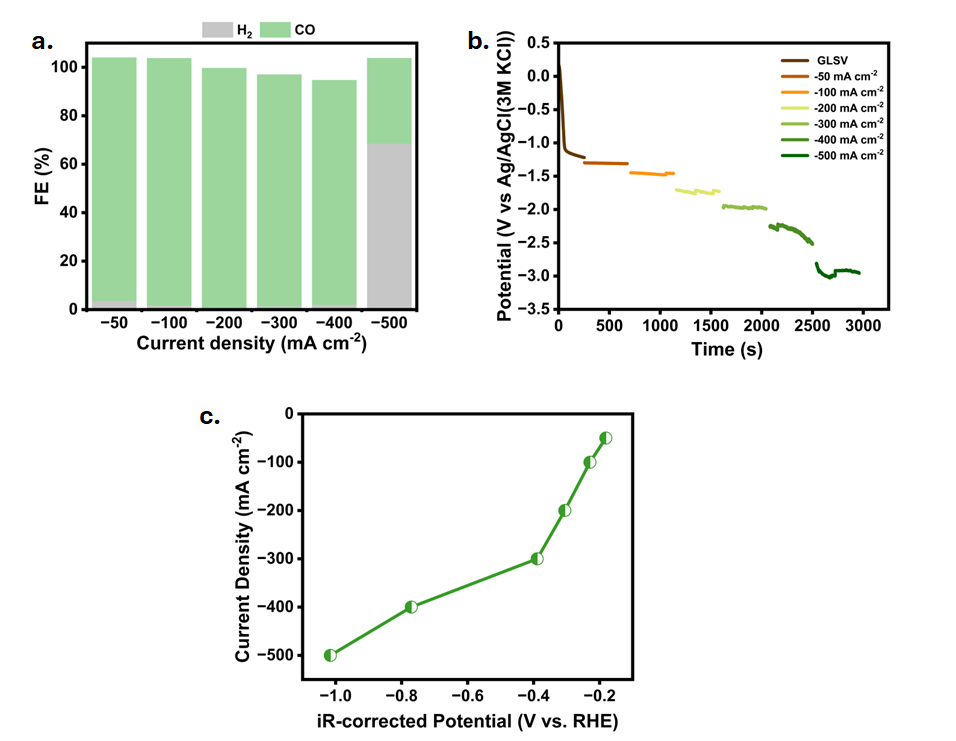


**Figure S14.** (a) FE, (b) uncompensated potential, (c) iR corrected potentials for a CuNi-SAC catalyst modified electrode at a CO_2_ flux of 15 ml min^-1^.


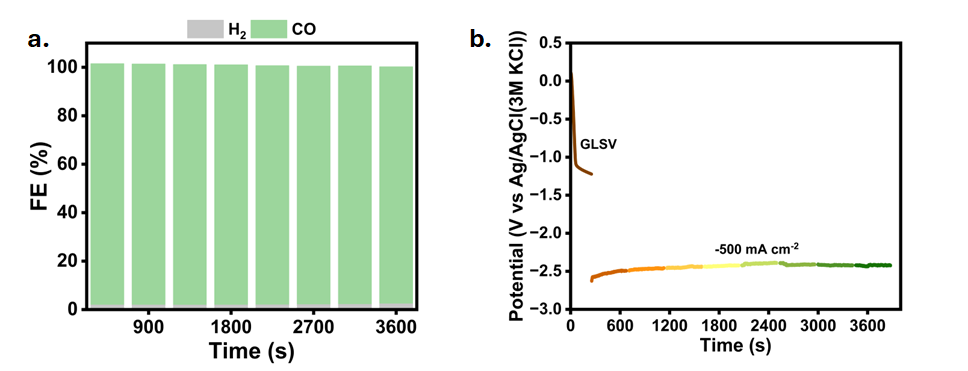
**Figure S15.** Long-term measurement at -500 mA cm^-2^. (a) FE, (b) applied potential for a CuNi-SAC modified GDE.


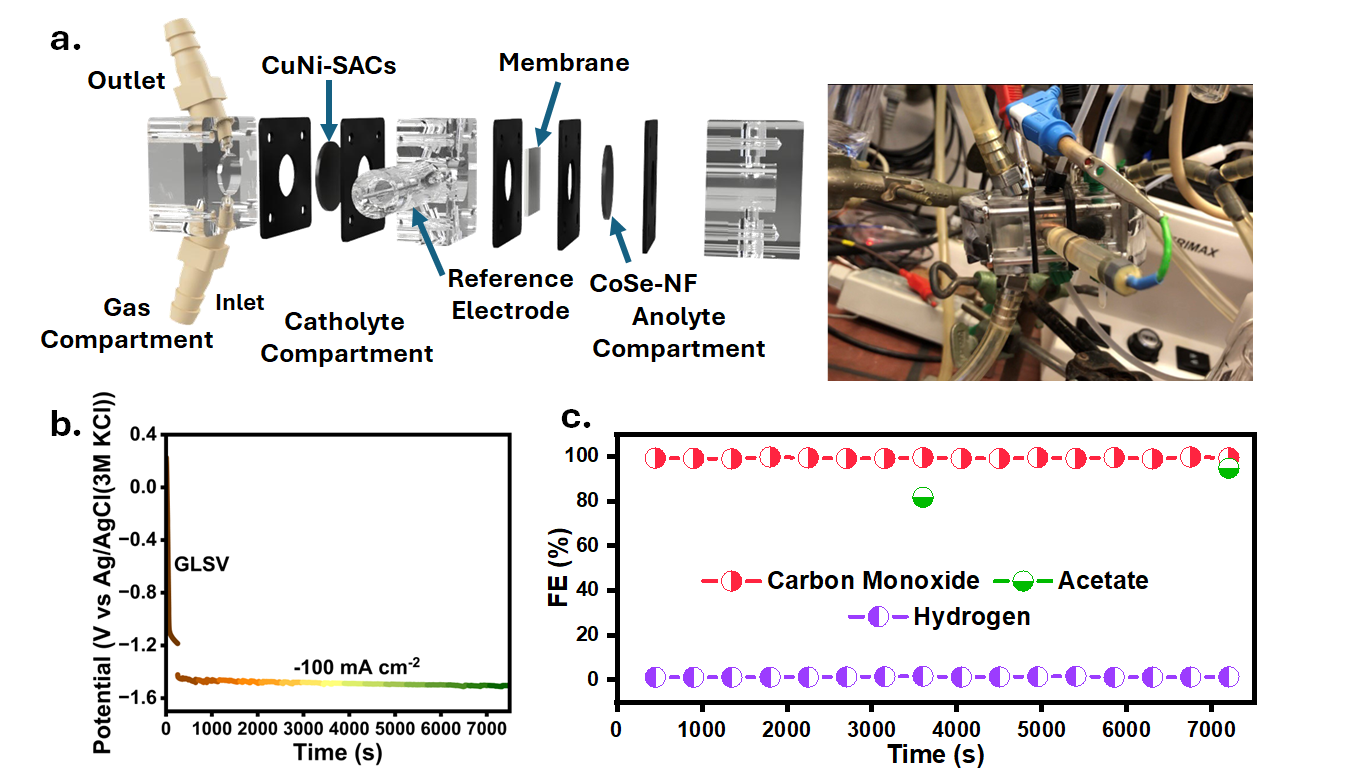


**Figure S16.** a) Schematic of the flow cell electrolyser used for coupled measurements eCO_2_RR and eEOR. b) Uncompensated potential during chronopotentiometry at -100 mA cm^-2^. c) FE for CO and acetate during coupled electrolysis at -100 mA cm^-2^.


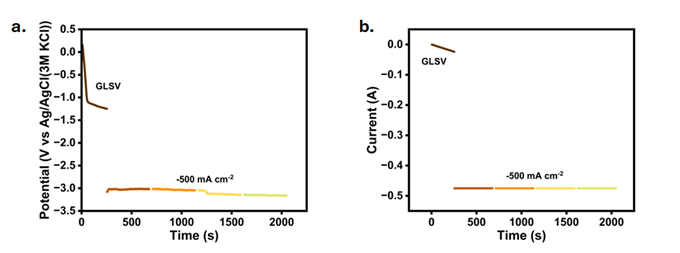
**Figure S17.** (a) Uncompensated potential at -500 mA cm^-2^, (b) applied currents for cell-1 in the tandem cell setup.


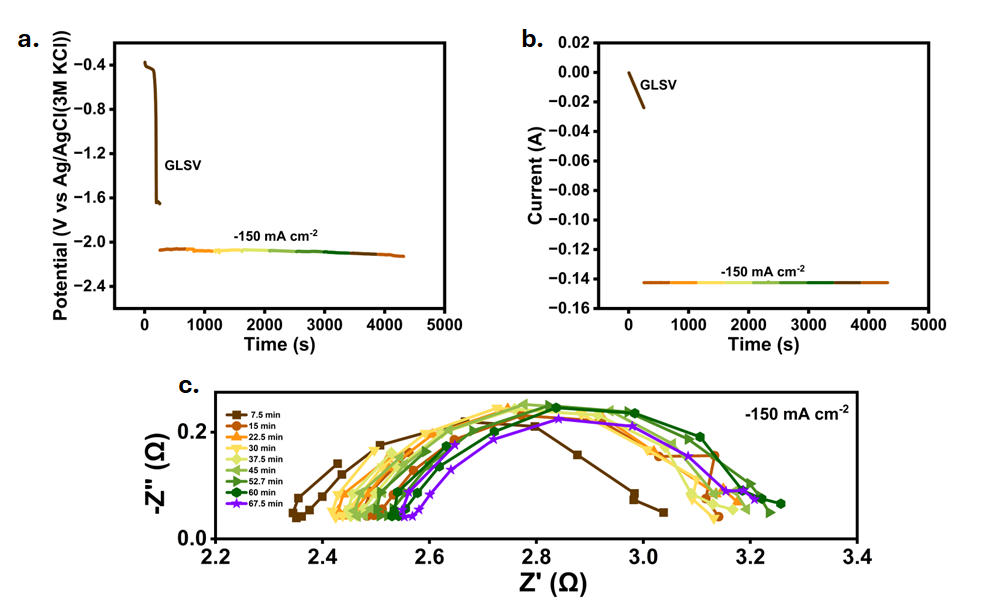
**Figure S18**. a) Uncompensated potential, (b) applied currents, and (c) GEIS measurements for the long term experiment at -150 mA cm^-2^ for cell-2 in the tandem cell setup.


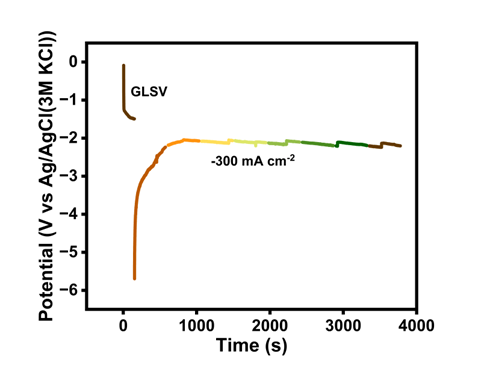


**Figure S19**. Uncompensated potential for cell-1 at -300 mA cm^-2^ during the coupled tandem cell measurement.


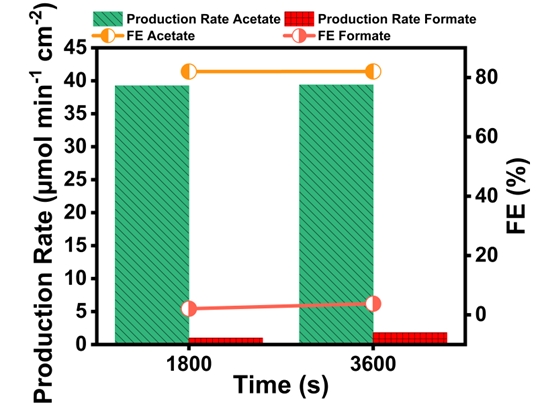


**Figure S20.** Production rate and FE in long-term coupled electrolysis measurements for cell-1 (CuNi-SAC as cathode (1 M KOH) and CoSe/NF as anode (1 M KOH + 1 M EtOH)) at -300 mA cm^-2^.


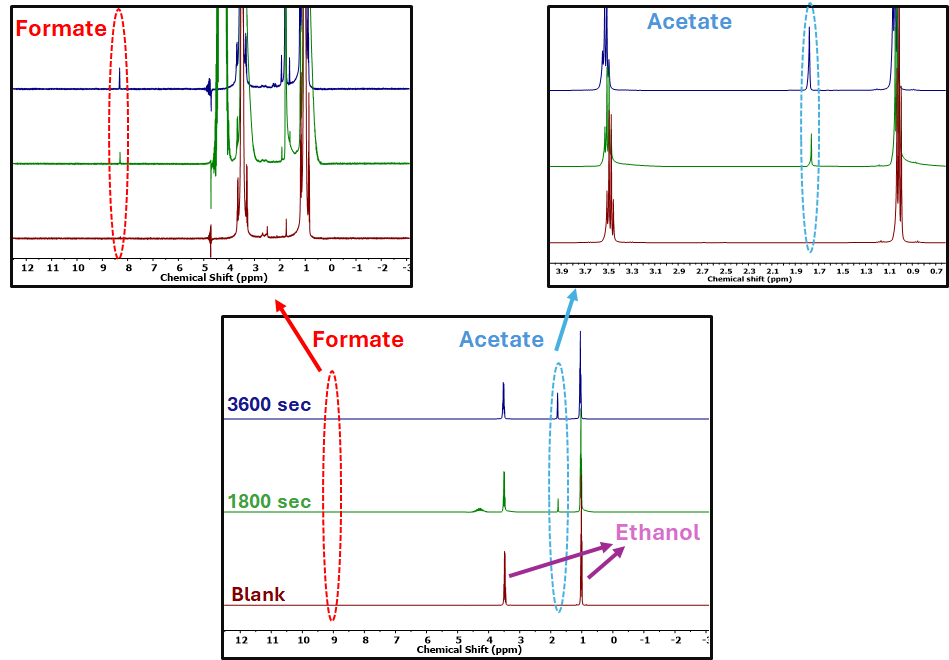


**Figure S21.** ^1^H-NMR spectra of the electrolyte of cell-1 recorded every 1800 s.

**Single electrode reactions -**

Electrochemical ethanol oxidation on CoSe/NF (denoted as M) here,

CH_3_CH_2_OH + M → M-(OCH_2_CH_3_)_ads_ + H^+^ + e^-^ (Adsorption)

M-(OCH_2_CH_3_)_ads_ → M-(OCHCH_3_)_ads_ + H^+^ + e^-^ (Dehydrogenation)

(At this stage, acetaldehyde can leave the electrode surface and dissolve in the electrolyte)

M + OH- → M-OH_ads_ + e^-^ (Hydroxide adsorption)

CH_3_CHO_ads_ → M- (COCH_3_)_ads_ + H^+^ + e^-^ (Dehydrogenation)

M-(COCH_3_)_ads_ + M-OH_ads_ →M-(CH_3_COOH)_ads_ (Rate-Determining Step)

M-(CH_3_COOH)_ads_ + OH^-^ → CH_3_COO^-^ + H_2_O + M (Desorption)

**C_2_H_5_OH + 5OH^-^ → CH_3_COO^-^ + 4H_2_O +4e^-^ (Pathway for Ethanol to Acetate)**

These are critical steps for acetate formation; the adsorbed acetyl intermediate (CH_2_CO_ads_) needs to add an oxygen atom. The oxygen comes directly from the adsorbed hydroxide species (OH)_ads_ on the catalyst's surface, through nucleophilic attack on the intermediate, rapidly converting to acetate.^[3]^ If the pH is low, the low OH^-^ concentration can cause the acetyl intermediate to stick on the catalyst surface, potentially poisoning the catalyst. We targeted acetate as a product from eEOR, which requires high pH conditions; otherwise, the product can lead to the formation of acetaldehyde.


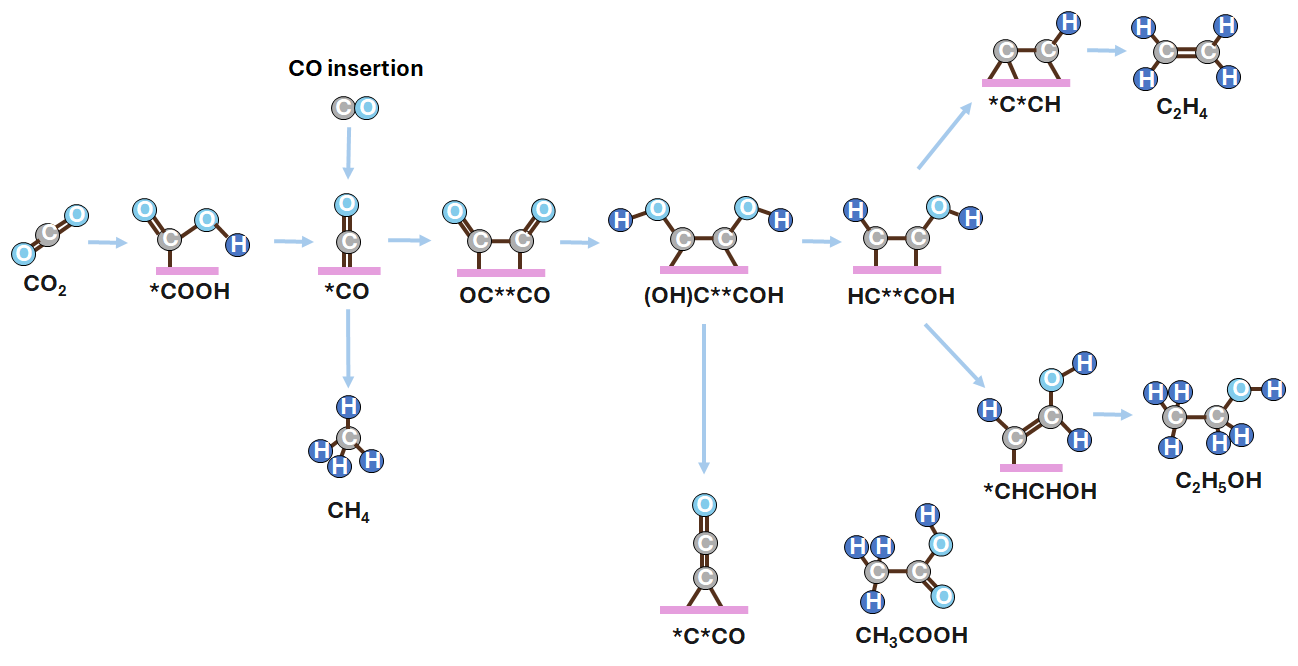


**Figure S22.** Possible mechanistic pathways for eCO_2_RR products.^[1]^

**Electrochemical CO_2_ reduction**

CuNi-SAC (for CO) and D-Cu-triazole (for acetate) which are denoted as M here,

CO_2_ + M → M-(CO_2_)_ads_

M-(CO_2_)_ads_ + H_2_O + e^-^ → M-(COOH)_ads_ + OH^-^ (CO_2_ activation)

M-(COOH)_ads_ + H_2_O +e^-^ → M-(CO)_ads_ + H_2_O + OH^-^ (Formation of *CO, surface contain 2 *CO)

M-(CO)_ads_ + M-(CO)_ads_ + H_2_O + e^-^ → M-(OCCOH)_ads_ + OH^-^ (Reductive coupling)

M-(OCCOH)_ads_ + e^-^ → M-(CCO)_ads_ + OH^-^ (OH removal)

M-(CCO)_ads_ + 2H_2_O + 2e^-^ → M-(CH_2_CO)_ads_ + 2OH^-^ (Hydrogenation)

M-(CH_2_CO)_ads_ + OH^-^ → M+CH_3_COO^-^ (Chemical Step)

**2CO_2_ + 5H_2_O + 8e^-^ → CH_3_COO^-^ + 7OH^-^ (Pathway for eCO_2_RR to Acetate)**

Acetate production requires a high local pH because hydroxide ions act as a reactant in the mechanism. Without a high concentration of OH^-^, the reaction can proceed along either the ethanol or ethylene pathway. When the *CO coverage increases, C-C coupling between *CO and *CO (or *CHO) occurs, which is then reduced to (HCCOH)_ads_, leading to the formation of ethanol and ethylene. For the acetate pathway, (OCCOH)_ads_ can dehydrate to form a ketene intermediate, where the abundant OH^-^ ions at high pH act as a nucleophile and attack the central carbon of the ketene, forming acetate.^[4]^

**Table S1**. Accumulated concentration for acetate (mmol/L) detected by HPCL after every 450 s at each current density for half-cell eEOR.

| **Current Density (mA cm^-2^)** | **mmol/L detected from HPLC (Acetate)** |
| --- | --- |
| \| **100** \|  \| \| --- \| --- \| \| **200** \|  \| \| **300** \|  \| \| **400** \|  \| \| **500** \|  \| \| **600** \|  \| | \| 4.6 \| \| --- \| \| 13.6 \| \| 28.0 \| \| 47.8 \| \| 72.1 \| \| 91.8 \| |
| \| **200** \|  \| \| --- \| --- \| \| **400** \|  \| \| **600** \|  \| \| **700** \|  \| \| **800** \|  \| | \| 7.9 \| \| --- \| \| 23.6 \| \| 44.8 \| \| 66.4 \| \| 89.7 \| |
| \| **200** \|  \| \| --- \| --- \| \| **1000** \|  \| \| **1000** \|  \| | \| 8.2 \| \| --- \| \| 37.2 \| \| 63.9 \| |

**Table S2**. Accumulated concentration for acetate (mmol/L) detected by HPCL after every 900 s at -150 mA cm^-2^ used for coupled tandem cell measurement.

| **Time** | **mmol/L detected from HPLC (Acetate)** |
| --- | --- |
| \| **900 s** \| \| --- \| \| **1800 s** \| \| **2700 s** \| | \| 0.57 \| \| --- \| \| 1.26 \| \| 2.02 \| |

**References**

[1] A. Shekhawat, D. Das, R. Zerdoumi, M. A. A. Mahbub, B. Eid, S. Chandra, S. Seisel, W. Schuhmann, "Defect-induced selectivity modulation using copper triazole molecular frameworks for electrochemical CO_2_ reduction." *Adv. Funct. Mater.* **2025**, *35,* 2506172.

[2] G. Lu, X. Wang, J. Timoshenko, B. R. Cuenya, G. Zhao, X. Huang, W. Schuhmann, M. Muhler, "A 3D macroporous carbon NiCu single-atom catalyst for high current density CO_2_ electrore­duction." *Adv. Funct. Mater.* **2025**, *35,* 2419075.

[3] M. Wala, W. Simka, " Effect of Anode Material on Electrochemical Oxidation of Low Molecular Weight Alcohols - A Review." *Molecules* **2021**, *26(8), 2144*.

[4] A. Shekhawat, M. A. A. Mahbub, W. Schuhmann, " Electrochemical CO_2_ Reduction toward Acetate/Acetic Acid." *ChemElectroChem* **2026**, *13,* e202500413.
